# A novel lncRNA uc.134 represses hepatocellular carcinoma progression by inhibiting CUL4A-mediated ubiquitination of LATS1

**DOI:** 10.1186/s13045-017-0449-4

**Published:** 2017-04-19

**Authors:** Wen Ni, Yuqin Zhang, Zetao Zhan, Feng Ye, Yonghao Liang, Jing Huang, Keli Chen, Longhua Chen, Yi Ding

**Affiliations:** 1 Department of Radiation Oncology, Nanfang Hospital, Southern Medical University, Guangzhou, 510515 China; 2 Department of Pathology, Southern Medical University, Guangzhou, 510515 China; 3 Cancer Center, Affiliated Hospital of Guangdong Medical University, Zhanjiang, 524001 China

**Keywords:** Hepatocellular carcinoma, LncRNA uc.134, CUL4A, Ubiquitination, LATS1, pYAP^S127^

## Abstract

**Background:**

Hepatocellular carcinoma (HCC) is one of the most common malignancies worldwide, and tumor recurrence and metastasis are major factors that contribute to the poor outcome of patients with HCC. Long noncoding RNAs (lncRNAs) are known to regulate different tumorigenic processes, and a growing body of evidence indicates that Hippo kinase signaling is inactivated in many cancers. However, the upstream lncRNA regulators of Hippo kinase signaling in HCC are poorly understood.

**Methods:**

Using a lncRNA microarray, we identified a novel lncRNA, uc.134, whose expression was significantly decreased in the highly aggressive HCC cell line HCCLM3 compared with MHCC97L cells. Furthermore, we evaluated uc.134 expression in clinical samples using in situ hybridization (ISH) and quantitative real-time polymerase chain reaction (qRT-PCR) analysis. The full-length transcript of uc.134 was confirmed using rapid amplification of cDNA ends (RACE) analyses. To investigate the biological function of uc.134, we performed gain-of-function and loss-of-function studies both in vitro and in vivo. The underlying mechanisms of uc.134 in HCC were investigated using RNA pulldown, RNA immunoprecipitation, ubiquitination assays, Western blotting, mRNA microarray analyses, and qRT-PCR analyses.

**Results:**

The ISH assay revealed that uc.134 expression was significantly decreased in 170 paraffin-embedded samples from patients with HCC compared with adjacent tissues and uc.134 expression directly correlated with patient prognosis. Furthermore, we defined a 1867-bp full-length transcript of uc.134 using 5′- and 3′-RACE analysis. The overexpression of uc.134 inhibited HCC cell proliferation, invasion, and metastasis in vitro and in vivo, whereas the knockdown of uc.134 produced the opposite results. Furthermore, we confirmed that uc.134 (1408–1867 nt) binds to CUL4A (592–759 aa region) and inhibits its nuclear export. Moreover, we demonstrated that uc.134 inhibits the CUL4A-mediated ubiquitination of LATS1 and increases YAP^S127^ phosphorylation to silence the target genes of YAP. Finally, a positive correlation between uc.134, LATS1, and pYAP^S127^ was confirmed in 90 paraffin-embedded samples by ISH and immunohistochemical staining.

**Conclusions:**

Our study identifies that a novel lncRNA, uc.134, represses hepatocellular carcinoma progression by inhibiting the CUL4A-mediated ubiquitination of LATS1 and increasing YAP^S127^ phosphorylation. The use of this lncRNA may offer a promising treatment approach by inhibiting YAP and activating Hippo kinase signaling.

**Electronic supplementary material:**

The online version of this article (doi:10.1186/s13045-017-0449-4) contains supplementary material, which is available to authorized users.

## Background

Hepatocellular carcinoma (HCC) is one of the most common causes of cancer mortality worldwide [1, 2]. Currently, surgical treatment for HCC is only available in the initial stage of the disease, but most patients present with advanced disease upon diagnosis, at which point the efficacy of radiotherapy and chemotherapy is limited. The high rate of tumor recurrence and metastasis are major factors that contribute to the poor prognosis of patients with HCC. Therefore, novel insights into the mechanism of HCC are urgently needed to identify novel prognostic molecular markers and potential effective therapeutic targets to improve patient survival [3, 4].

Long noncoding RNAs (lncRNAs) are RNA transcripts that are longer than 200 nt and exhibit limited or no protein-coding capacity, and many lncRNAs are uniquely expressed in differentiated tissues or specific cancer types [5–8]. Recent discoveries indicate that lncRNAs drive many important cancer phenotypes by interacting with other cellular macromolecules, including DNA, RNA, and protein [9–12]. Advanced studies have found that aberrant lncRNA expression plays critical roles in hepatocarcinogenesis and metastasis [13–15]. Moreover, MALAT1 is associated with tumor metastasis and can predict recurrence after liver transplantation [16, 17]. Furthermore, UFC1 promotes HCC cell proliferation by inhibiting cell apoptosis and inducing cell cycle progression [18]. Long noncoding RNA low expression in tumor (lncRNA-LET) reduces hepatic invasion and abdominal metastases via the degradation of NF90 [19], and MEG3 is a predictive biomarker for monitoring epigenetic therapy [20]. In addition, ultraconserved noncoding RNAs (ucRNAs) are noncoding RNAs transcribed from regions that are highly conserved across many species, so-called ultraconserved regions [21]. Evf-2, a ucRNA in the intergenic region between the Dlx-5 and Dlx-6 genes, activates transcriptional activity by directly interacting with Dlx-2 in a target- and homeodomain-specific manner [22]. However, the molecular mechanisms of lncRNAs remain poorly understood and warrant further study.

A growing number of studies have focused on the widespread Hippo kinase signaling inactivation and nuclear localization of YAP in epithelial malignancies [23–25]. Specifically, a gene expression analysis of HCC identified YAP as an important “driver oncogene” [26], and the Hippo pathway is a highly conserved protein kinase chain that plays a pivotal role in restricting tumor cell proliferation and promoting apoptosis. By phosphorylating YAP, the serine/threonine-protein kinase LATS1 inhibits the translocation of YAP into the nucleus and decreases the expression of its downstream target genes, which are important for cell proliferation and migration [27]. However, the mechanism by which lncRNAs regulate Hippo kinase signaling in HCC remains largely unclear.

In this study, we used a lncRNA microarray to identify that the expression of the ultraconserved lncRNA uc.134 was significantly decreased in the highly aggressive HCC cell line HCCLM3 compared with MHCC97L cells. We then first confirmed the full-length transcript of uc.134 using 5′- and 3′-rapid amplification of cDNA ends (RACE) analysis (GenBank accession no. KY355383). The high conservation of uc.134 implicates that its aberrant expression may play a critical role in HCC progression. Moreover, in situ hybridization (ISH) and quantitative real-time polymerase chain reaction (qRT-PCR) results showed that uc.134 expression significantly decreased in HCC and uc.134 expression directly correlated with patient survival. Furthermore, we demonstrated that the overexpression of uc.134 suppresses HCC cell proliferation and invasion by inhibiting CUL4A to ubiquitinate LATS1 and increasing pYAP^S127^ expression. We also confirmed that uc.134 inhibits the expression of YAP downstream target genes. Finally, a positive relationship among uc.134, LATS1, and pYAP^S127^ was also confirmed in 90 paraffin-embedded clinical samples. In conclusion, our study reveals that the novel lncRNA uc.134 represses HCC progression by inhibiting the CUL4A-mediated ubiquitination of LATS1 and increasing pYAP^S127^ expression. Thus, this lncRNA may offer a promising treatment approach by inhibiting YAP and activating Hippo kinase signaling.

## Methods

### Microarray assay

Total RNA was isolated from cells using TRIzol Reagent (Takara, Dalian, China). Sample labeling and array hybridization were performed according to the Arraystar microarray-based gene expression analysis protocol (Arraystar, Rockville, MD). The Arraystar human lncRNA microarray is designed for the global profiling of human lncRNAs and protein-coding mRNA transcripts. The array detects a total of 40,173 lncRNAs. The lncRNAs were carefully constructed using the most highly respected public transcriptome databases (RefSeq, UCSC Known Genes, Ensembl, etc.), as well as landmark publications. The Arraystar mRNA microarray provided a global view of all known genes and transcripts in the human genome. A total of 27,958 Entrez Gene RNAs were detected by this microarray. The content was sourced from RefSeq, Ensembl, UniGene Build, and GenBank. Quantile normalization and subsequent data processing were performed using the GeneSpring GX v12.1 software package (Agilent Technologies). After quantile normalization of the raw data, the lncRNAs and mRNAs that were detected in at least three out of six samples were chosen for further data analysis. Differentially expressed lncRNAs and mRNAs with statistical significance between the two groups were identified through *P* value/FDR filtering. KEGG pathway analysis and gene ontology (GO) analysis were applied to determine the roles of these differentially expressed mRNAs in the corresponding biological pathways or GO terms. The microarray data were uploaded in the Additional files 1, 2, and 3.

### Tissue samples, ISH, immunohistochemical staining (IHC), and fluorescence in situ hybridization (FISH)

From January 2009 to December 2013, 170 human HCC samples were collected at Nanfang Hospital, Southern Medical University (Guangzhou, China). None of these patients had been pretreated with chemotherapy or radiotherapy before undergoing surgery. The study was approved by the Nanfang Hospital Institutional Ethical Review Board, and informed consent was obtained from each patient.

LncRNA uc.134 expression was measured in paraffin-embedded samples using an ISH optimization kit (Roche, Basel, Switzerland) according to the manufacturer’s instructions. The locked nucleic acid (LNA)-modified oligonucleotide probe targeting uc.134 was designed and synthesized at Exiqon (Vedbaek, Denmark). Briefly, HCC samples were treated with pepsin for 10 min at room temperature and incubated with 500 nM of probe at 55 °C for 4 h. The samples were incubated with blocking solution for 30 min, anti-digoxigenin (anti-DIG) reagent was applied for 60 min and the samples were incubated with AP substrate 4-nitro-blue tetrazolium and 5-bromo-4-chloro-3′-indolylphosphate (NBT-BCIP) for 2 h at 30 °C. The samples were then mounted with Nuclear Fast Red™ (BOSTER, Wuhan, China), and a blue stain in the nucleus indicated a positive signal by NBT-BCIP. IHC was performed as we previously described [28]. For FISH, the signals representing the expression of LNA probes were determined using the tyramide signal amplification (PerkinElmer, USA) system. In brief, the signal was detected by incubation with horseradish peroxidase (HRP)-conjugated anti-DIG antibodies. Then, the signals were amplified using tetramethylrhodamine (TRITC)-conjugated tyramide. The images were acquired with a fluorescence microscope (IX70, Olympus, Japan).

The ISH and IHC results were evaluated by two individuals in a blinded fashion; the evaluators scored the samples using a quick scoring system from 0 to 12 by combining the intensity and percentage of the positive signal (signal: “0,” no staining; “1,” weak staining; “2,” intermediate staining; and “3,” strong staining; percentage: “0,” 0%; “1,” 1–25%; “2,” 26–50%; “3,” 51–75%; and “4,” >75%), and this was in good agreement with the initial quantification. An optimal cutoff value was identified. If the evaluated uc.134 score was higher than the average score, the uc.134 expression in those HCC samples was classified as high; otherwise, it was classified as low. To account for inconsistencies in the percentage of the ISH signals, an ImageJ software (National Institutes of Health, Bethesda, MD) was used for scoring signals. The data were statistically analyzed using *t* test to determine the differences in uc.134 expression levels between different groups of tissues. *P* < 0.05 was considered significant.

### In vivo model

All animal studies were performed with the approval from the Institutional Animal Care and Use Committee of Nanfang Hospital. Male BALB/c nude mice (age, 4–6 weeks; Guangdong Medical Laboratory Animal Center, China) were raised under specific pathogen-free conditions. All in vivo experiments were performed according to our institution’s guidelines for the use of laboratory animals. For the subcutaneously injected tumor model, 3 × 10^6^ cells were subcutaneously injected into the left flanks or right flanks of mice. After 4 weeks, the tumors were embedded in paraffin for ISH or IHC. For the lung metastasis model, 2 × 10^6^ cells were injected into the tail veins. We monitored lung metastasis at 6 weeks to quantify lung colonization by histology examination.

### RACE analyses

The 5′ and 3′-RACE experiments were performed using the SMARTer® RACE 5′/3′ Kit (Clontech, Mountain View, CA) according to the manufacturer’s instructions. Briefly, at least two sets of primers were designed and synthesized for the nested PCR. The RACE PCR products were separated on a 1.5% agarose gel. The results of electrophoresis were confirmed, and the amplified bands were sequenced bi-directionally using the indicated primers. The gene-specific RACE primers used for mapping each end are listed in Additional file 4.

### RNA isolation and qRT-PCR analysis

Cytoplasmic and nuclear RNA fractionation was performed using the PARIS™ Kit (Life Technologies, Carlsbad, California) according to the manufacturer’s instructions. The yield and quality of the RNA samples were evaluated prior to qRT-PCR. For qRT-PCR analysis, total RNA was isolated from the cells using TRIzol Reagent (Takara) following the manufacturer’s protocol. First-strand cDNA synthesis from 1 μg of total RNA was performed using a reverse transcriptase cDNA synthesis kit (Takara). The resulting cDNA was then analyzed by qRT-PCR using a SYBR Green PCR Kit (Takara) and a 7500 Fast real-time PCR system (AB Applied Biosystems). In brief, the reaction mixture containing 500 ng cDNA, the forward primer and the reverse primer, was used to amplify the PCR product corresponding to the human gene. The experiments were repeated at least three times independently to ensure the reproducibility of the results. Human GAPDH gene was amplified as an internal control. β-actin and U6 were used as cytoplasmic and nuclear controls, respectively. Comparative quantification was done by using the 2^−ΔΔCt^ method. The primer sequences are listed in Additional file 4.

### RNA immunoprecipitation (RIP)

A RIP assay was performed using the Magna RIP RNA-Binding Protein Immunoprecipitation Kit (Millipore, MA, USA) according to the manufacturer’s instructions. Briefly, whole-cell extracts prepared in lysis buffer containing a protease inhibitor cocktail and RNase inhibitor were incubated on ice for 5 min, followed by centrifugation at 10,000*g* and 4 °C for 10 min. Magnetic beads were preincubated with 5 ug of IP-grade antibody for 30 min at room temperature with rotation. The supernatant was added to bead-antibody complexes in immunoprecipitation buffer and incubated at 4 °C overnight. Finally, the RNA was purified and quantified by qRT-PCR. Input controls and normal rabbit IgG controls were assayed simultaneously to ensure that the signals were detected from RNA that was specifically bound to protein.

### RNA pulldown assay

Biotin-labeled RNA uc.134 was transcribed in vitro with the Biotin RNA Labeling Mix (Roche) and T7 RNA polymerase (Roche) and then treated with RNase-free DNase I (Roche) and 0.2 M EDTA to stop the reaction. Biotinylated RNAs were mixed with streptavidin agarose beads (Life Technologies, Gaithersburg, MD) at 4 °C overnight. Total cell lysates and RNase inhibitor were added to each binding reaction and incubated on ice for 1 h. The RNA–protein binding mixture was boiled in SDS buffer, and the eluted proteins were detected by Western blotting or mass spectrometry. The full-length transcript of uc.134 is 1867 bp in length; Δ1, Δ2, and Δ3 correspond to the 1–718 bp, 719–1407 bp, and 1408–1867 bp sequence fragments of uc.134 until the end of the uc.134 sequence. CUL4A was cloned into the eukaryotic expression vector pcDNA3.1(+) with a C-terminal Myc tag and translated a 87.7-kilodalton (kDa) protein. CUL4A lacking the 55–401 amino acid (aa) region was cloned into pcDNA3.1(+) to afford the pcDNA3.1(+)-Cul4a-△1-myc construct, which translated a 46.94-kDa protein; CUL4A lacking the 400–671 aa region encoding a cullin homolog was cloned into pcDNA3.1(+) to afford the pcDNA3.1(+)-Cul4a-△2-myc construct, which translated a 56.53-kDa protein; CUL4A lacking the 688~753 aa region, which encodes a neddylation domain, was cloned into pcDNA3.1(+) to afford the pcDNA3.1(+)-Cul4a-△3-myc construct, which translated a 79.81-kDa protein; CUL4A lacking the 592–759 aa region, which encodes the winged helix-turn-helix DNA-binding domain (WHDD), was cloned into pcDNA3.1(+) to afford the pcDNA3.1(+)-Cul4a-△4-myc vector, which translated a 68.03-kDa protein.

### Cycloheximide (CHX) chase measurements of LATS1 half-life

The CUL4A and uc.134 plasmids were transiently transfected into HCC cells using jetPRIME (Polyplus, Strasbourg, France). After 24 h, CHX (10 ug/ml) was added to the DMEM culture medium, and incubation was continued for 0, 3, 6, or 9 h. The cell lysates were submitted to Western blotting using rabbit anti-LATS1 monoclonal antibody (Cell Signaling Technology, Beverly, MA), and Western blot data were quantified using the ImageJ software.

### Immunoprecipitation (IP) and ubiquitination assay

Ubiquitin, LATS1, and uc.134 plasmids were transfected into cells using jetPRIME (Polyplus). Thirty-six hours after transfection, 10 nM MG132 was added to the DMEM culture medium and incubation was continued for 8 h. The lysates were immunoprecipitated with the indicated antibodies on protein A/G beads (Life Technologies) overnight at 4 °C with rotation and then boiled in SDS buffer. The eluted proteins were detected by Western blotting.

### Statistical analysis

All statistical analyses were performed using the SPSS software (Chicago, IL, USA). Survival curves were plotted based on the Kaplan–Meier and log-rank tests. Pearson’s chi-square test was used to analyze the relationship between uc.134 expression and the clinicopathologic features of HCC. Student’s *t* test was used to detect significance differences in data obtained from qRT-PCR experiments and colony formation assays. A multi-way classification analysis of variance test was performed to assess data obtained from the CCK8 assays and tumor growth. Correlations among uc.134 expression, LATS1, and pYAP^S127^ were analyzed with a Spearman rank correlation. *P* < 0.05 was considered to indicate a significant difference.

## Results

### Decreased lncRNA uc.134 expression in HCC patients significantly correlates with poor survival

**Fig. 1:**
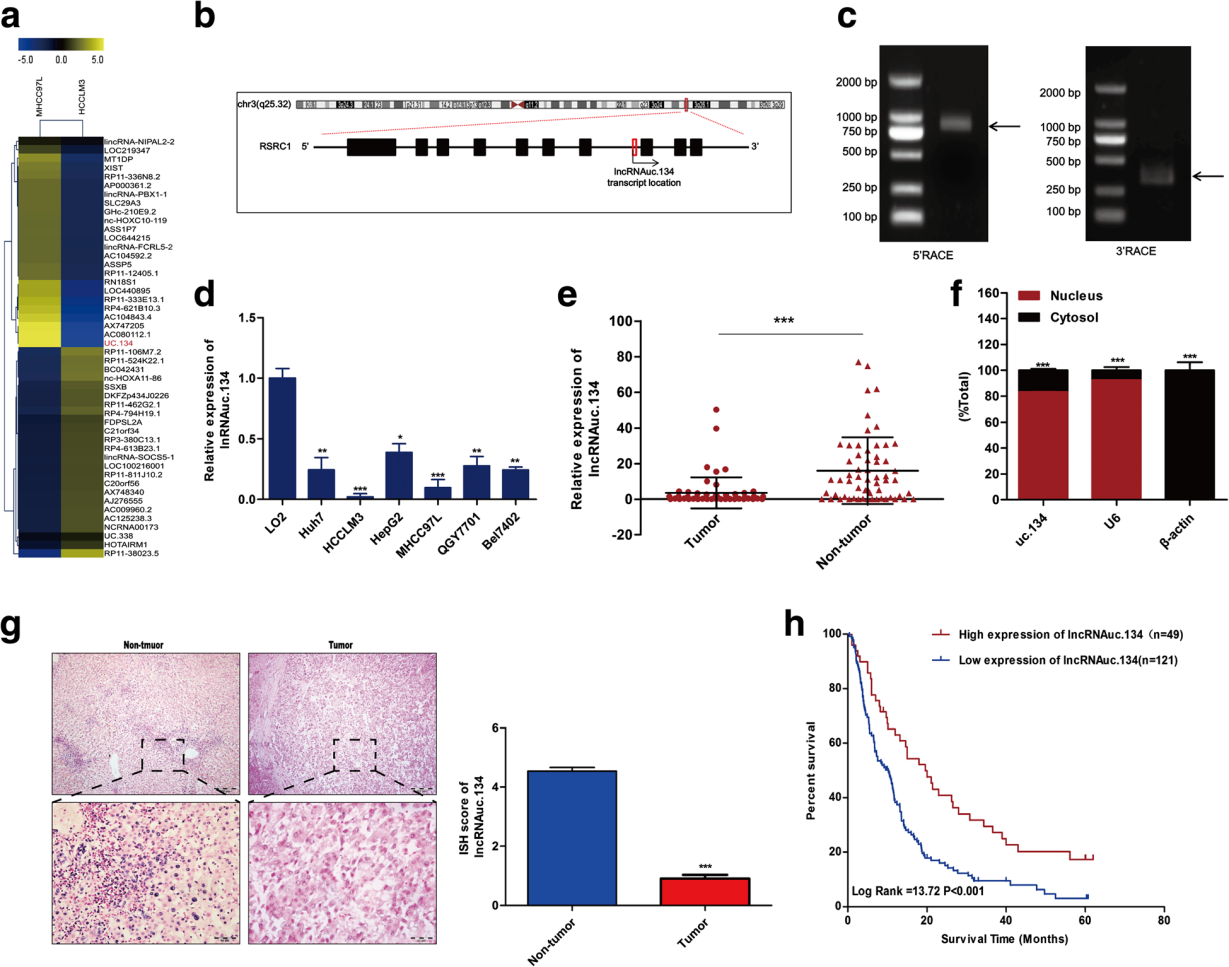
Aberrant expression of lncRNA uc.134 in HCC. **a** Hierarchical clustering analysis of lncRNAs differentially expressed in HCCLM3 and MHCC97L HCC cells. Upregulated genes are highlighted in *yellow*, and downregulated genes are in *blue*. (fold changes >5; *P* < 0.05). **b** Transcript of uc.134. **c** PCR products from the 5′-RACE and 3′-RACE procedures as shown by agarose gel electrophoresis. **d**–**e** The expression of uc.134 was analyzed by qRT-PCR in HCC tissues and HCC cells. *P < 0.05; **P < 0.01; and ***P < 0.001. **f** qRT-PCR analysis of RNAs purified from nuclear (*red*) and cytosolic (*black*) compartments in Bel7402 cells. ***P < 0.001. **g** ISH assays of uc.134 expression in HCC paraffin specimens and adjacent nontumor tissues. ***P < 0.001. **h** The Kaplan–Meier method was used to assess the survival of patients with HCC, and the log-rank test was used to compare survival between the uc.134-low and lncRNAuc.134-high groups (*n* = 170)

Previous studies have shown that lncRNA expression profiles are significantly altered in HCC. The authors of a previous study reported that they isolated a parent human HCC cell line MHCC97 and its two derived cell lines with different metastatic potentials (MHCC97-L, with low metastatic potential, and MHCC97-H, with high metastatic potential) [29]. MHCC97-H was inoculated subcutaneously into nude mice, and the HCCLM3 cell line was established from lung metastasis in the mice. These HCC cell lines have the same genetic background and yet have dramatically different metastatic behavior [30]. To identify HCC metastasis-relevant lncRNAs, we compared the expression profiles of lncRNAs between the highly aggressive cell line HCCLM3 and the weakly aggressive cell line MHCC97L using human lncRNAs microarray (Fig. 1a). A total of 1050 upregulated lncRNAs and 611 downregulated lncRNAs with significantly differential expression (≥3.0-fold) were identified. To validate our findings from the microarray analysis, the top four upregulated and three downregulated known/novel lncRNAs were detected using qRT-PCR in HCC cell lines and the immortalized hepatocyte cell line LO2 and the results demonstrated significant differential expression trends similar to those predicted by the microarray (Additional file 5: Figure S1). Among these lncRNAs, the ultraconserved noncoding RNA uc.134 whose transcript is located in the intron region of the RSRC1 gene (Fig. 1b) exhibited the greatest downregulation in both tissue samples and HCC cell lines (Fig. 1d, e). We used 5′- and 3′-RACE analyses to identify a 1867-bp full-length transcript of uc.134 (Fig. 1c, Additional file 5: Figure S2). By analysis of cytoplasmic and nuclear RNA fractionation and FISH experiments from HCC cells, we observed that the expression of uc.134 was relatively high in the nucleus (Fig. 1f, Additional file 5: Figure S3). ISH analysis was used to determine expression of uc.134 in 170 paraffin-embedded surgical specimens of HCC from Nanfang Hospital. The expression of uc.134 was significantly downregulated in the tumor tissues compared with the adjacent tissues (*P* < 0.001) (Fig. 1g, Fig. 6a). Forty-nine of tumor tissues (28.8%) exhibited a high expression of uc.134, and the other 121 cases (71.2%) had low expression and the Kaplan–Meier analysis indicated that the lower uc.134 expression was related to poor overall survival in patients with HCC (*P* < 0.001). The median survival time of the HCC patients with low uc.134 expression was 10 months, which was significantly shorter than the survival time of those with high uc.134 expression (20 months) (Fig. 1h). Moreover, an analysis of the clinical follow-up data combined with clinicopathologic parameters demonstrated that the expression of uc.134 significantly correlated with lymph node metastasis (*P* = 0.005) and tumor node metastasis (TNM) classification (*P* < 0.001) of patients with HCC (Table 1).

**Table 1:** Relationship between lncRNA uc.134 expression and the clinical characteristics of HCC patients

lncRNA uc.134 expression					
Features	*n*	Low	High	*x* ^2^	*P* value
All cases	170	121	49		
Age				0.169	0.412
<50	56	41	15		
≥50	114	80	34		
Gender				0.332	0.377
Man	145	102	43		
Female	25	19	6		
Histological grade				2.488	0.288
Well	13	8	5		
Moderately	79	53	26		
Poorly	78	60	18		
HBsAg status				0.001	0.566
Negative	48	34	14		
Positive	119	84	35		
Serum AFP (ng/ml)				3.885	0.037
<25	67	42	25		
≥25	103	79	24		
Tumor size (cm)				0.042	0.487
<5	113	81	32		
≥5	57	40	17		
Tumor number				4.044	0.032
Single	145	99	46		
Multiple	25	22	3		
Lymph node metastasis				6.851	0.005
Negative	139	93	46		
Positive	26	24	2		
TNM stage				51.393	0.000
I and II	67	27	40		
III and IV	103	94	9		

*P* value <0.05 was considered to indicate statistical significance. The *P* values were calculated in SPSS 17.0 using Pearson’s chi-square test

### LncRNA uc.134 inhibits HCC cell proliferation, invasion, and metastasis in vitro and in vivo

**Fig. 2:**
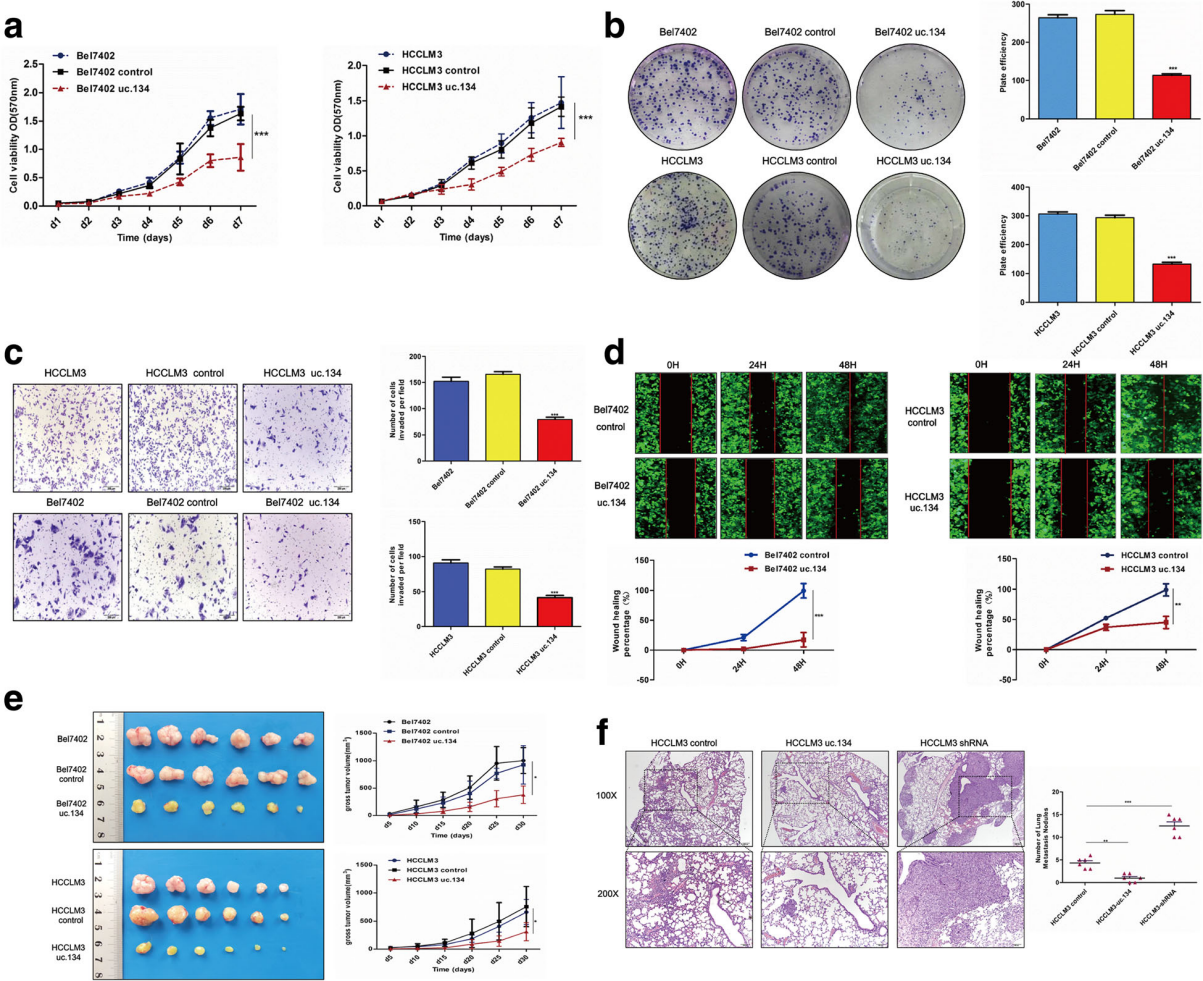
LncRNA uc.134 suppresses the proliferation, migration, and invasion of HCC in vitro and in vivo. **a** Growth curves of indicated HCC cells that overexpressed uc.134 compared with those of negative control cells and untransfected cells were determined with a CCK8 proliferation analysis. The mean ± SD is shown for five independent experiments. ***P < 0.001. **b** Representative images of colony formation assays (*left panels*); analysis of the number of colonies (*right panels*). All experiments were performed in triplicate, and results are presented as mean ± SD. ***P < 0.001. **c** Invasion ability of the indicated *cell lines* by Transwell assays. All experiments were performed in triplicate, and results are presented as mean ± SD. ***P < 0.001. **d** Representative images of the scratch wound-healing assay. The wound-healing percentage was analyzed with the ImageJ software. All experiments were performed in triplicate, and results are presented as mean ± SD. **P < 0.01 and ***P < 0.001. **e** Representative images of tumors formed in nude mice (*n* = 6). *P < 0.05. **f** Lung metastasis model generated by injecting tumor cells into the tail veins of mice. HE staining showing the number and volume of lung metastases in each group (*n* = 6); **P < 0. 01 and ***P < 0.001

To investigate the biological significance of uc.134 in the progression of HCC, we performed gain-of-function and loss-of-function studies in vitro and in vivo. The results of CCK8 proliferation assays and colony formation assays showed that the stable overexpression of EGFP-LV-uc.134 (Additional file 5: Figure S4A) significantly reduced the proliferative capacity of HCC cells and that cells overexpressing uc.134 formed fewer colonies than control cell lines containing the empty vector (Fig. 2a, b). Transwell and scratch wound-healing assays showed that the upregulation of uc.134 markedly reduced cell invasion compared with control cells (Fig. 2c, d), whereas the knockdown of uc.134 by EGFP-LV-shRNA (Additional file 5: Figure S4B) promoted HCC cell growth and invasion (Additional file 5: Figure S5A–S5D).

For in vivo studies, we generated a xenograft tumor model by subcutaneously injecting HCC cells in which uc.134 was stably overexpressed or knocked down into the flanks of nude mice. The overexpression of uc.134 markedly decreased the tumor growth rate and mean tumor volume compared with the negative control group (Fig. 2e), whereas silencing uc.134 produced the opposite results (Additional file 5: Figure S5E). Moreover, ISH, IHC, and hematoxylin-eosin (HE) staining were performed on paraffin-embedded samples of xenograft tumors formed by cells overexpressing EGFP-LV-uc.134 or EGFP-LV-shRNA and the negative control cells. The results showed that in the xenograft tumors overexpressing uc.134, the level of KI67 expression was lower than that in the negative control, whereas in the uc.134 silenced tumors, the level of KI67 expression was higher (Additional file 5: Figure S6). We further generated a tail vein xenograft tumor model by injecting the indicated cells into the tail veins of nude mice. At the end of the experiment, the lungs of the mice were removed for HE staining to histologically examine lung metastases. LncRNA uc.134-overexpressing cells showed reduced lung colonization ability compared with controls, whereas the knockdown of uc.134 produced the opposite results (Fig. 2f, Additional file 5: Figure S5F). Briefly, our studies confirmed that uc.134 can inhibit HCC cell proliferation and invasion, whereas uc.134 knockdown promotes HCC growth and metastasis, suggesting that lncRNA uc.134 may have a critical role in suppressing the development of HCC.

### Association of lncRNA uc.134 with CUL4A

**Fig. 3:**
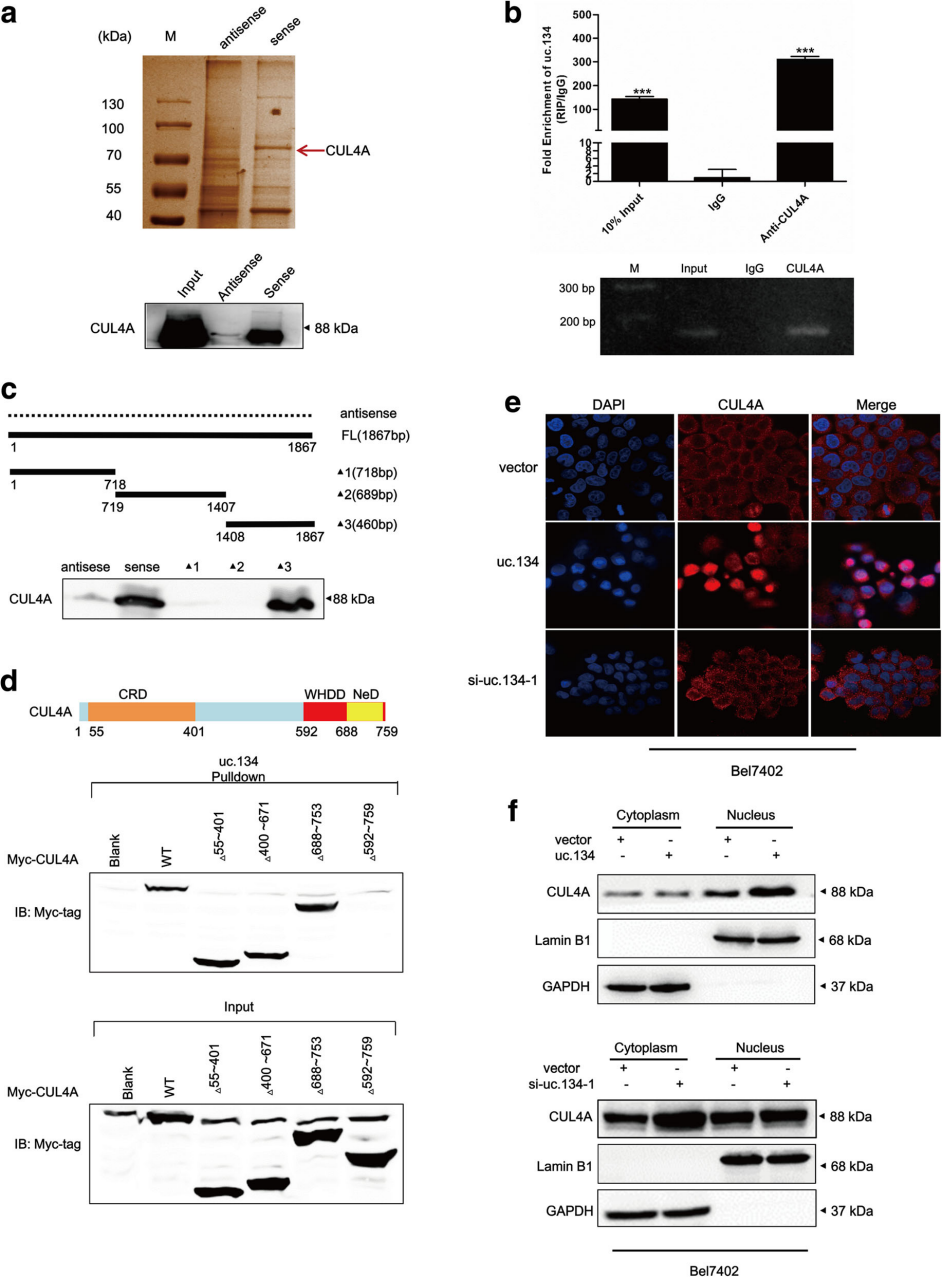
LncRNA uc.134 binds CUL4A and inhibits CUL4A nuclear export. **a** LncRNA uc.134-associated proteins identified by RNA pulldown and mass spectrometric analyses in HEK293T cells (*upper panel*). Western blot analysis of pulldown product (*bottom panel*). **b** RIP assays in HEK293T cells. qRT-PCR analysis of RIP (*upper panel*). Agarose electrophoresis of PCR products (*bottom panel*). Experiments were performed in triplicate, and data are presented as mean ± SD. ***P < 0.001. **c** Immunoblot (IB) detection of CUL4A retrieved by in vitro transcribed biotinylated RNAs corresponding to different fragments of uc.134 or its antisense sequence (*dotted line*) in HEK293T cells. **d** IB detection of Myc-tagged CUL4A (WT versus domain truncation mutants) precipitated by in vitro transcribed biotinylated uc.134 in HEK293T cells. *Upper panel*: graphic illustration of the domain structure of CUL4A. **e**–**f** LncRNA uc.134 inhibits CUL4A nuclear export as demonstrated by immunofluorescence staining (**e**) and Western blotting (**f**)

Recently, several studies have focused on the molecular regulation networks between lncRNAs and proteins. To explore the molecular mechanisms of uc.134 in HCC progression, we performed RNA pulldown assays to identify proteins associated with uc.134. After 10% SDS polyacrylamide gel electrophoresis and silver staining, the differentially expressed bands were identified by comparison with the antisense control and excised and subjected to mass spectrometry. An analysis of RNA-associated proteins in three independent RNA pulldown assays followed by mass spectrometry detected the E3 ubiquitin ligase CUL4A, and subsequent Western blot analyses confirmed these results (Fig. 3a). We also performed RIP to verify the candidate proteins pulled down with uc.134 using antibodies against CUL4A, and a nonspecific antibody (IgG) was used as a control. The data showed that CUL4A directly bound to uc.134 (Fig. 3b). Taken together, these results confirmed that uc.134 and CUL4A can form an RNP complex in vitro. Previous studies have shown that lncRNAs can function as unstructured sequences and the functions of these RNAs may depend more on their linear sequences than their conserved secondary structures [31]. To identify the unique binding sites, we took advantage of a series of deletion mutants of uc.134 to map the CUL4A-binding region: Δ1 corresponds to the region between nucleotides 1–718 of uc.134, which is the truncated 5′ end; Δ2 corresponds to the region between nucleotides 719–1407; and Δ3 corresponds to the region between nucleotides 1408–1867 of uc.134, which is the truncated 3′ end. The results from in vitro binding assays indicated that CUL4A interacted with a 460-nt region at the 3′ end of uc.134 (Δ3-1408–1867) (Fig. 3c). Protein domain mapping studies demonstrated that uc.134 binds the 592–759 amino acid (aa) region of CUL4A (Fig. 3d). The 592–759 aa region of CUL4A encodes a domain known as the winged helix-turn-helix DNA-binding domain (WHDD), which has been suggested to bind DNA. This suggestion is consistent with our observation that the WHDD of CUL4A may serve as the RNA-binding domain for uc.134. Taken together, these results demonstrate that uc.134 directly interacts with CUL4A to form uc.134-CUL4A RNPs.

CUL4A is amplified in various cancers, which suggests its role in regulating cell cycle progression, transcription, and embryonic development. At present, numerous proteins have been reported to be ubiquitinated by CUL4A including p53, p21, p27, DICER, and LATS1 [32]. Previous studies have shown that inflammation-induced Jak-STAT3 signaling triggers the ubiquitination of DICER1 in cytoplasm by CUL4A to promote the development of colon cancer [33]. Although CUL4A is predominantly located in the cytoplasm, a fraction of CUL4A protein resides in the nucleus and mediates nucleosome reassembly [34–36]. Thus, we performed Western blotting and immunofluorescence analyses to investigate the mechanism by which uc.134 regulates CUL4A expression. The knockdown or upregulation of uc.134 did not change total CUL4A protein expression. Interestingly, an analysis of immunofluorescence staining suggested that CUL4A protein accumulated in the nucleus when uc.134 was overexpressed, whereas uc.134 knockdown arrested CUL4A protein in the cytoplasm (Fig. 3e). The cytoplasmic and nuclear protein fractions from HCC cells revealed the same phenomenon (Fig. 3f).

### LncRNA uc.134 inhibits the CUL4A-mediated ubiquitination of LATS1 to regulate Hippo kinase signaling

**Fig. 4:**
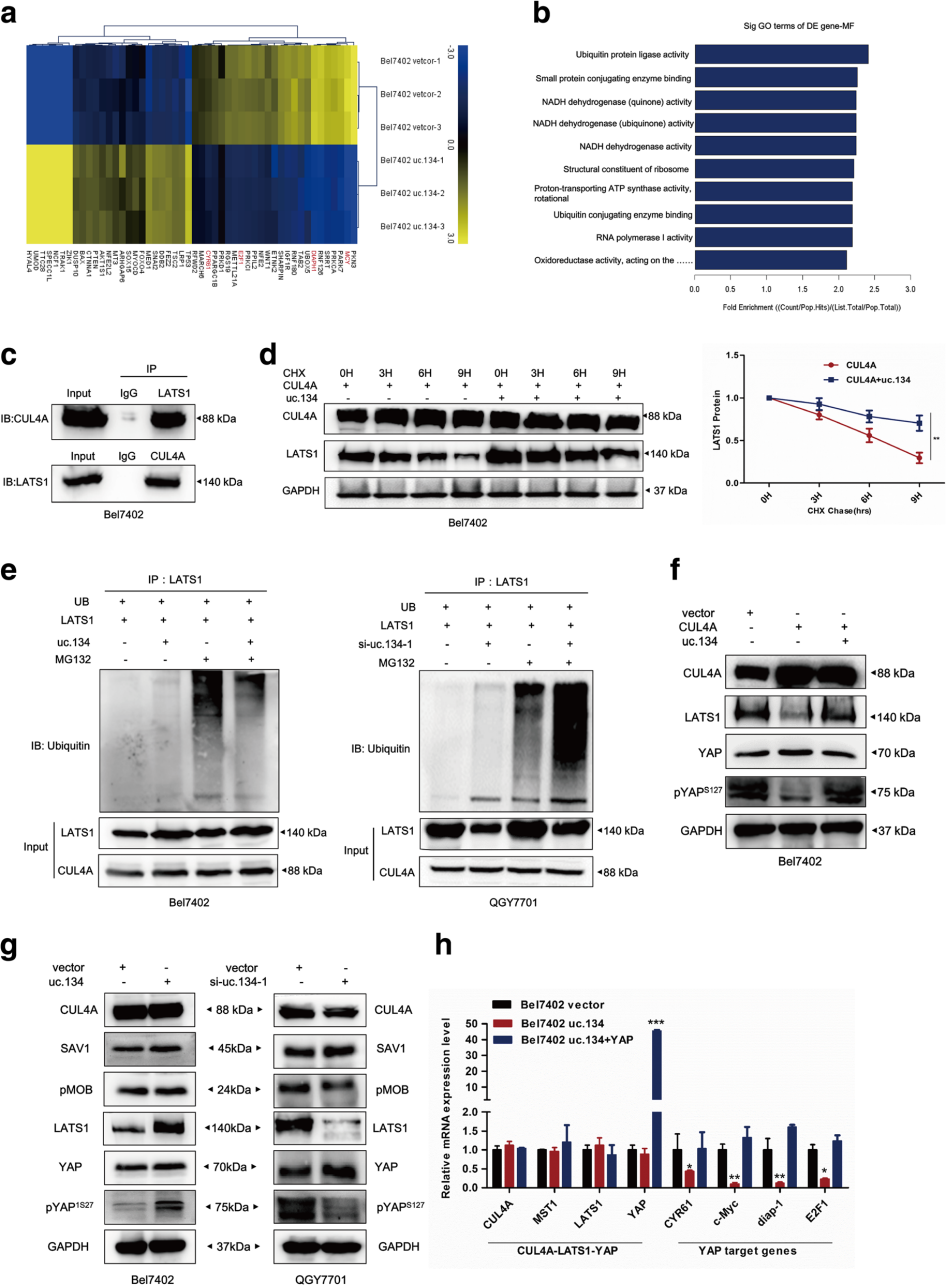
LncRNA uc.134 inhibits the CUL4A-mediated ubiquitination of LATS1 to regulate Hippo kinase signaling. **a**–**b** Hierarchical clustering analysis (**a**) and GO analysis (**b**) of microarray data for HCC cells overexpressing uc.134 and control cells. Upregulated genes are highlighted in *yellow*, and downregulated genes are in *blue*. (fold changes >3; *P* < 0.05). The top ten most significantly enriched GO terms in differentially expressed genes relative to all other genes in the genome are shown. **c** IP experiment. **d** The overexpression of uc.134 prolonged the half-life of LATS1. Immunoblot detection of LATS1 (*left*); IB data were quantified using the ImageJ software (*right*). *Error bars* indicate the mean ± SD. **P < 0.01. **e** Ubiquitination assays of cells transfected with uc.134 plasmid in Bel7402 cells (*left*) or siRNA in QGY7701 cells (*right*). The *bottom panels* depict the input of the cell lysates. **f** Western blotting in Bel7402 cells showed that uc.134 reversed the CUL4A-mediated inhibition of Hippo kinase activity. **g** Western blots showed that the overexpression of uc.134 activates Hippo kinase signaling (*left*), whereas silencing of uc.134 yielded opposite results (*right*). **h** qRT-PCR analysis of cells overexpressing uc.134 or co-transfected with YAP plasmids compared with vector controls. Experiments were performed in triplicate, and data are presented as the mean ± SD. *P < 0.05; **P < 0.01; and ***P < 0.001

The data from the mRNA microarray and GO analyses showed that the overexpression of uc.134 significantly repressed ubiquitin protein ligase and RNA polymerase activity compared with the negative control (Fig. 4a, b). Previous studies showed that the Hippo pathway core component LATS1 contains an N-terminal ubiquitin-binding domain that can bind in cis or trans to C-terminal ubiquitinated sequences, inducing conformational changes that disturb kinase activity [37]. Since CUL4A is an E3 ligase that targets LATS1 protein for ubiquitination and degradation, we performed an immunoprecipitation assay using either anti-LATS1 antibody or anti-CUL4A antibody to precipitate CUL4A or LATS1, respectively. The results confirmed the interaction between endogenous LATS1 and CUL4A protein in HCC cells (Fig. 4c). Although a large number of studies demonstrate that LATS1 plays essential roles in the Hippo pathway, which suppresses tumorigenesis and stem cell differentiation, the association between LATS1 and lncRNA molecular regulators remains largely unknown. Thus, we also performed a CHX chase assay to assess the ability of uc.134 to enhance LATS1 protein stability. The results showed that LATS1 protein was much more stable in cells transfected with both CUL4A and uc.134 compared with cells transfected with CUL4A alone (Fig. 4d). We subsequently performed a ubiquitination assay, which showed a significant decrease in polyubiquitinated LATS1 protein in uc.134-transfected cells, whereas the knockdown of uc.134 increased LATS1 ubiquitination (Fig. 4e). Previous studies indicated that polyubiquitylation promotes LATS1 protein degradation and blocks its kinase activity to phosphorylate YAP [37]. Moreover, we performed a Western blot analysis, which showed that uc.134 overexpression reversed the CUL4A-mediated decrease in LATS1 expression and inactivation of Hippo kinase signaling (Fig. 4f).

Furthermore, microarray analysis and qRT-PCR indicated that the overexpression of uc.134 did not significantly affect the mRNA levels of members of the Hippo signaling pathway, such as Mob1, MST1/2, LATS1, and YAP, whereas the mRNA levels of YAP target genes, such as cysteine-rich angiogenic inducer 61 (CYR61), c-Myc, drosophila inhibitor of apoptosis 1 (diap1), and E2F1, were significantly reduced. In addition, we have performed the rescue experiment to test whether the uc.134-mediated changes in the tumorigenesis genes are dependent on YAP. The Bel7402 cells were transfected with the uc.134 overexpressing plasmid alone or together with the YAP overexpressing plasmid for 48 h, and the expression of target genes was evaluated by qRT-qPCR. The results showed that there was no reduction in the expression of CYR61, c-Myc, diap1, and E2F1 induced by uc.134 overexpression in the YAP-upregulated cells, indicating that YAP is responsible for the uc.134-mediated gene regulation (Fig. 4h). Moreover, a Western blot analysis demonstrated that the overexpression of uc.134 upregulated LATS1 protein expression and YAP phosphorylation at Ser127 but had no effect on other critical proteins of the Hippo kinase signaling pathway. In contrast, the LATS1 and pYAP^S127^ levels were decreased in specimens in which uc.134 expression was silenced, indicating that LATS1 and pYAP^S127^ are downstream effectors of uc.134 (Fig. 4g). Taken together, these studies suggest that lncRNA uc.134 inhibits CUL4A translocation from the nucleus to the cytoplasm to disrupt polyubiquitin chains, which activates the Hippo kinase signaling and silences target genes of YAP by increasing the stability of LATS1 protein.

### LncRNA uc.134 represses HCC proliferation and metastasis via the uc.134-CUL4A-Hippo axis

**Fig. 5:**
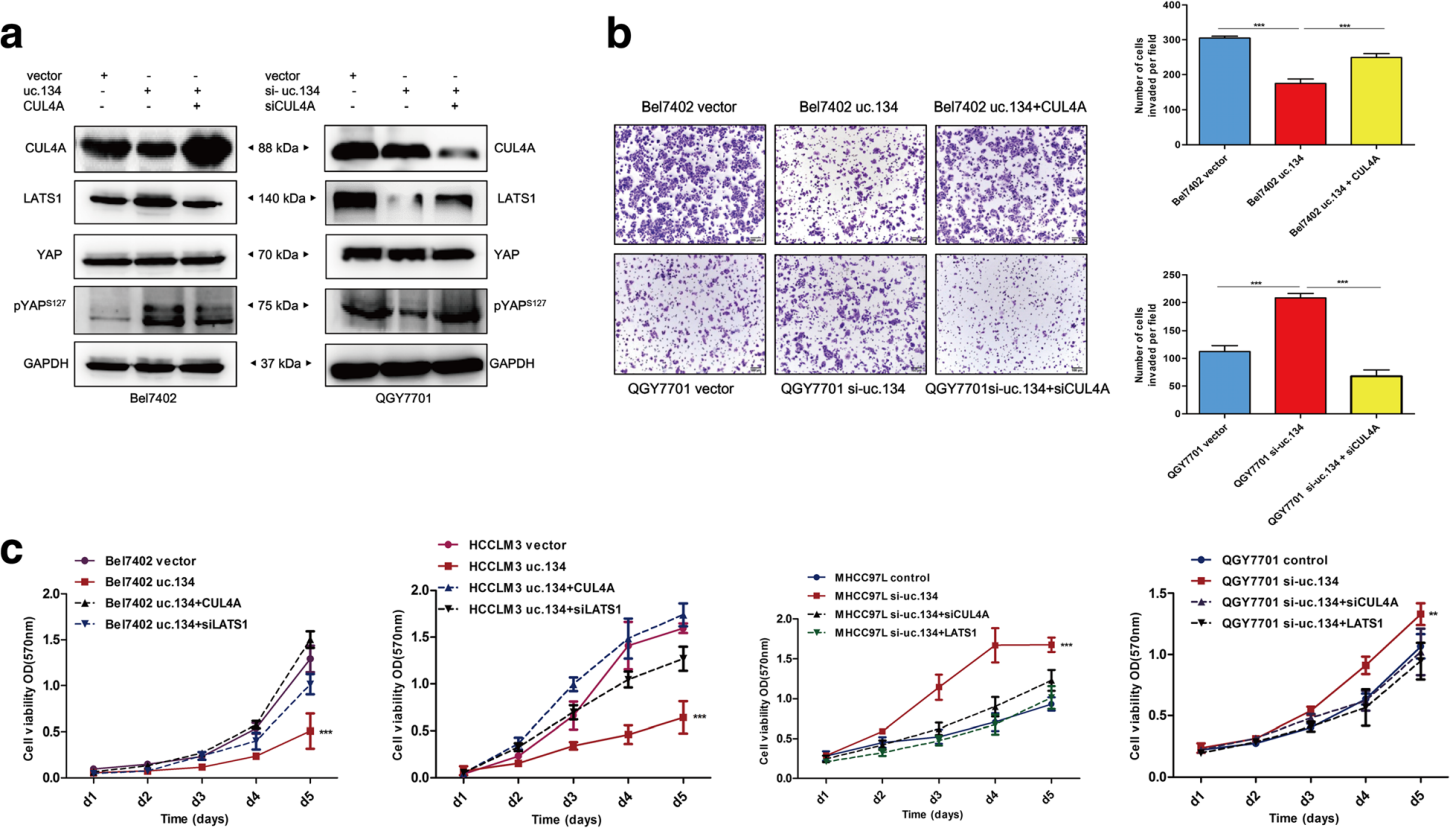
LncRNA uc.134 represses HCC proliferation and metastasis via the uc.134-CUL4A-Hippo axis. **a** Western blot showing that CUL4A abrogated the uc.134-mediated activation of Hippo kinase signaling (*left*), whereas the knockdown of CUL4A reversed the si-uc.134-mediated repression of LATS1 and inactivation of YAP (*right*). **b** A Transwell analysis showed that CUL4A overexpression abrogated the uc.134-mediated repression of HCC cell invasion (*upper*), whereas CUL4A knockdown yielded the opposite results (*lower*). All experiments were performed in triplicate, and results are presented as mean ± SD. ***P < 0.001. **c** A CCK8 proliferation analysis showed that the transfection of CUL4A plasmid or LATS1-specific siRNA abolished the uc.134-mediated growth inhibition (*left*), whereas the transfection of siCUL4A or LATS1 plasmid yielded opposite results (*right*). The mean ± SD is shown for five independent experiments. **P < 0.01; and ***P < 0.001

A Western blot analysis demonstrated that the overexpression of uc.134 upregulated LATS1 protein expression and YAP phosphorylation at Ser127, indicating that LATS1 and pYAP^S127^ are downstream effectors of uc.134. We then assessed the ability of CUL4A to overcome the uc.134-mediated activation of Hippo kinase signaling in HCC cells. Notably, Western blotting showed that the overexpression of CUL4A inhibited the uc.134-mediated increases in LATS1 and YAP^S127^ phosphorylation, whereas the knockdown of CUL4A reversed the si-uc.134-mediated repression of LATS1 and inactivation of Hippo kinase signaling (Fig. 5a, Additional file 5: Figure S4C). The biological function analysis showed that CUL4A overexpression abrogated the uc.134-mediated repression HCC cell proliferation and invasion, whereas CUL4A knockdown yielded the opposite results (Fig. 5b, c). Taken together, our data demonstrate that lncRNA uc.134 plays a key role in suppressing the proliferation, invasion, and metastasis of HCC by blocking CUL4A nuclear export to ubiquitinate LATS1 and increasing YAP^S127^ phosphorylation, which implicates the lncRNA uc.134-CUL4A-Hippo axis as a potential therapeutic target for the treatment of HCC.

### LncRNA uc.134 expression positively correlates with LATS1 and pYAP^S127^ levels in HCC patient samples

**Fig. 6:**
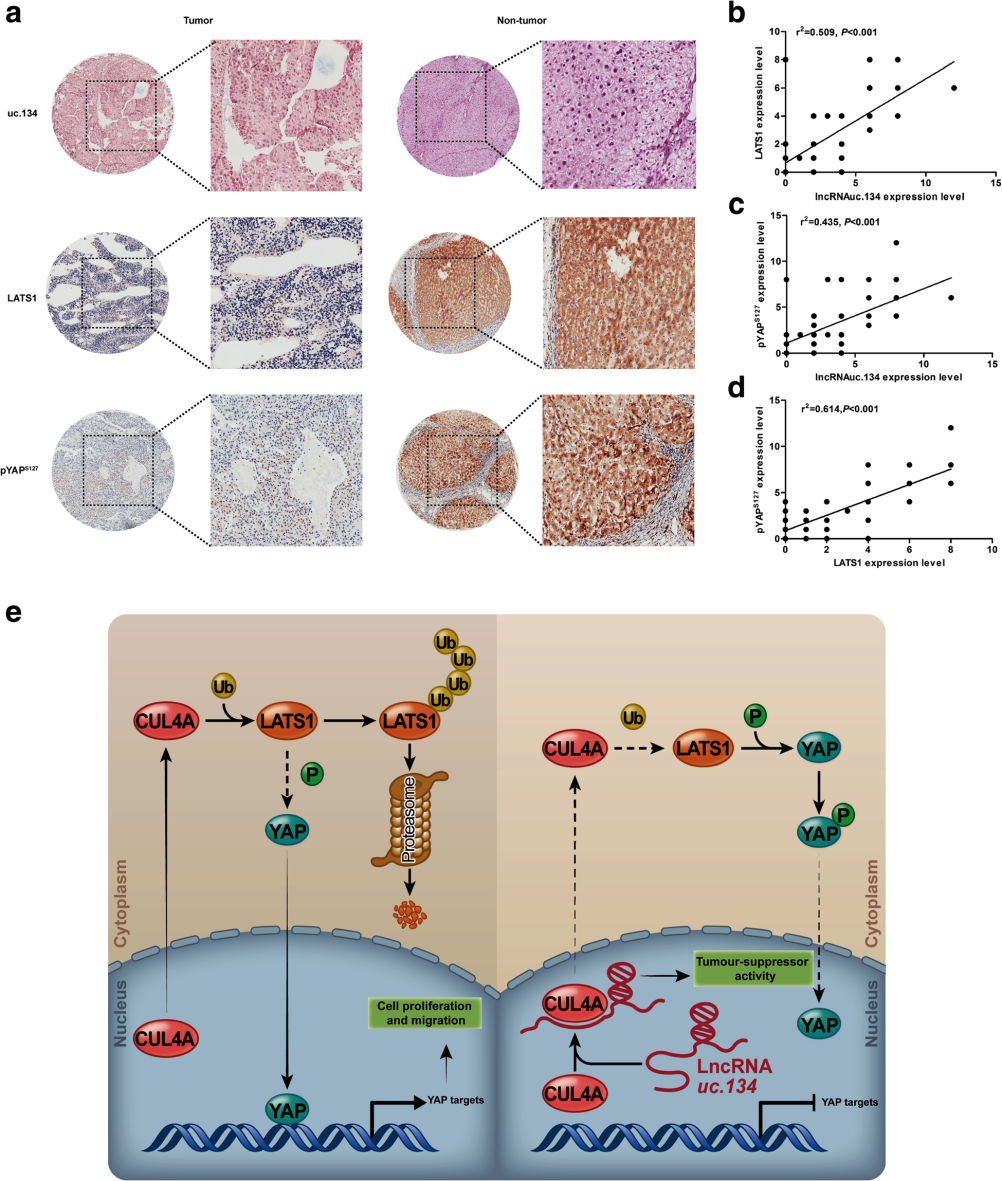
Expression levels of lncRNA uc.134, LATS1, and pYAP^S127^ positively correlated in samples from patients with HCC. **a** The ISH and IHC staining of HCC paraffin-embedded samples showed that the expression levels of uc.134, LATS1, and pYAP^S127^ were significantly downregulated in HCC (200× magnification, *n* = 90). **b**–**d** A scatter diagram showed positive correlations among uc.134 and LATS1 or pYAP^S127^. **e** A model for the regulatory mechanisms of uc.134 in HCC

We studied correlations among uc.134, LATS1, and pYAP^S127^ expression by ISH and IHC in paraffin-embedded samples from patients with HCC (Fig. 6a). IHC analysis was used to determine the expression of LATS1 or pYAP^S127^ in 90 paraffin-embedded surgical specimens of HCC from Nanfang Hospital. The expression of LATS1 was significantly downregulated in the tumor tissues compared with the adjacent tissues (*P* < 0.001) (Fig. 6a, Additional file 5: Figure S7A), and the Kaplan–Meier analysis indicated that the lower LATS1 expression was related to poor overall survival in patients with HCC (*P* = 0.032). The median survival time of the HCC patients with low LATS1 expression was 16 months, which was significantly shorter than those with high LATS1 expression (49 months) (Additional file 5: Figure S7C). In addition, the expression of pYAP^S127^ was also significantly downregulated in the tumors compared with the adjacent tissues (*P* < 0.01) (Fig. 6a, Additional file 5: Figure S7B). By the Kaplan–Meier analysis, it was observed that the low pYAP^S127^ protein level was a significant prognostic factor for poor overall survival in patients with HCC (*P* = 0.01). The median survival time of the HCC patients with low pYAP^S127^ expression was 16 months, which was significantly shorter than those with high pYAP^S127^ expression (50 months) (Additional file 5: Figure S7D). Together, these results clearly show that the expressions of LATS1 and pYAP^S127^ are reduced in the HCC tissues compared with the adjacent tissues, and low LATS1 or pYAP^S127^ expression is associated with worse prognosis. In addition, correlations among the expression of uc.134, LATS1, and pYAP^S127^ were analyzed with a Spearman’s rank correlation. The scatter plot showed positive relationships among uc.134, LATS1 (*r* ^2^ = 0.509; *P* < 0.001), and pYAP^S127^ (*r* ^2^ = 0.435; *P* < 0.001) and a positive relationship between LATS1 and pYAP^S127^ (*r* ^2^ = 0.614; *P* < 0.001) in 90 HCC specimens (Fig. 6b–d). Collectively, our data indicate that a 460-nt region of uc.134 (Δ3-1408–1867) binds to the WHDD (592–759 aa region) domain of CUL4A to inhibit CUL4A-mediated ubiquitination of LATS1 and activate Hippo kinase signaling. Thus, the lncRNA uc.134-CUL4A-Hippo axis may offer a promising approach for the treatment of HCC (Fig. 6e).

## Discussion

HCC is a leading cause of cancer-related death worldwide, and tumor recurrence and metastasis are major factors that contribute to the poor prognosis of patients with HCC. Moreover, recent research has greatly advanced our understanding of the essential role of lncRNAs in HCC [10, 12]. Although thousands of lncRNAs have been functionally characterized, the vast majority of members of this RNA class have not been thoroughly described. UcRNAs are noncoding RNAs transcribed from regions that are highly conserved across humans, mice, and rats, so-called ultraconserved regions (UCRs). UCRs are usually located at genomic regions that are involved in cancer and differentially expressed in carcinomas. Here, we used lncRNA microarrays to identify that the expression of an ultraconserved lncRNA, uc.134, was significantly decreased in the highly aggressive HCC cell line HCCLM3 compared with MHCC97L cells. We first confirmed the full-length transcript of uc.134 using 5′- and 3′-RACE analyses, and qRT-PCR showed that uc.134 expression was reduced in both tissue samples and HCC cell lines. Thus, the decreased expression of uc.134 in HCC, especially in highly aggressive cell lines, indicates that uc.134 may be a promising marker for HCC. ISH results showed that the expression of uc.134 was significantly downregulated in 170 paraffin-embedded HCC specimens and decreased uc.134 expression was related to poor survival in patients with HCC. Moreover, an analysis of clinical follow-up data and clinicopathologic parameters demonstrated that the expression of uc.134 significantly correlated with lymph node metastasis (*P* = 0.005) and TNM classification (*P* < 0.001) in patients with HCC.

Cancer is fundamentally a genetic disease with numerous alterations in DNA, RNA, and proteins that support tumor growth and development [38]. The molecular and cellular characteristics of cancer-associated lncRNAs are under distinct regulatory regimes that are different from physiological conditions [12]. Although numerous studies have used gene overexpression plasmids or lentiviral vectors to evaluate the functions of tumor-suppressor genes, the cellular toxicities of these gene carriers are inevitable. Therefore, a knockdown model such as RNAi is necessary. In our study, we performed gain-of-function and loss-of-function studies both in vitro and in vivo to demonstrate that uc.134 plays a critical role in the inhibition of cell proliferation, invasion, and metastasis in HCC. Ideally, Tet-Off and Tet-On systems will fulfill the requirements for the quantitative and temporal control of gene expression via nontoxic effector molecules, and these will be used in the future [39].

A growing number of studies confirm that the Hippo kinase signaling, which is critical for tumor progression and invasion, is inactivated in many cancers. The core components of Hippo kinase signaling pathway are conserved in mammals and have a complex network of crosstalk with other important signaling pathways, such as the TGFβ/SMAD, WNT/β-catenin, PI3-kinase/AKT, Hedgehog, Jak/Stat, and Notch pathways [40]. A previous study confirmed that YAP increases resistance to RAF- and MEK-targeted cancer therapies [41]. Moreover, the serine/threonine kinase LATS1 is a core kinase of Hippo kinase signaling pathway and plays important roles in tumor proliferation, apoptosis, and stem cell differentiation. A previous study showed that Merlin activates Hippo kinase signaling by inhibiting CRL4^DCAF1^, an E3 ubiquitin ligase of the CRL4 complex [42]. CUL4A is a core component of the CRL4 complex, and its N-terminus associates with a cullin-specific adaptor protein to recruit a large number of substrate proteins. CUL4A can interact with LATS1 protein and enhance its proteasomal degradation. Another study demonstrated that the de-repression of CRL4^DCAF1^ inhibited the activation of the Hippo pathway by directly binding to and ubiquitinating LATS1/2 in NF2-mutant tumors in the nucleus [37]. However, the mechanism that regulates LATS1 at the lncRNA level remains unknown. We performed RNA pulldown and RIP assays, which showed that uc.134 bound the E3 ligase CUL4A. Protein domain mapping and deletion mutation analyses identified a 592–759 aa region of CUL4A, which encodes a WHDD domain, that binds a 460-nt region at the 3′ end of uc.134 (Δ3-1408–1867). In addition, we demonstrated that uc.134 inhibited the translocation of CUL4A from the nucleus to the cytoplasm, which inhibits the CUL4A-mediated ubiquitination and degradation of LATS1 in the cytoplasm and increases YAP^S127^ phosphorylation. LncRNAs regulate many important activities in cancer through their interactions with DNA, RNA, and proteins. The mechanisms through which lncRNAs interact with proteins to regulate protein-protein interactions or modulate the subcellular transport of proteins are largely unknown [43]. It remains to be investigated whether the uc.134-CUL4A RNP can be retained in the nucleus through binding with other nuclear proteins or by forming an RNA–DNA triplex.

As a co-activator of transcription, YAP binds to the TEAD complex and elevates the levels of numerous target genes to promote tumor proliferation and metastasis. CYR61 is overexpressed in various cancers and has been reported to be involved in tumor growth and vascularization. Previous reports showed that the YAP-dependent expression of CYR61 increased the invasive activity of the glioblastoma cells [44]. Inactivation of c-Myc results in tumor dormancy and pluripotent differentiation of tumor cells. YAP has been reported to promote the transcriptional activity of c-Myc via interaction with c-Abl in HCC [45]. diap1 directly binds to and inhibits caspases to suppress apoptosis. Activation of YAP increased the transcription of diap1, cell proliferation, and tissue overgrowth [46, 47]. A previous study has shown that Yorkie promotes the overgrowth of drosophila neuroepithelial cells and delays their differentiation through the regulation of the cell cycle regulator E2F1, which plays key roles in cell cycle progression and cell differentiation in HCC [48, 49]. We performed microarray analysis and qRT-PCR indicated that overexpression of uc.134 significantly decreased the expression of YAP target genes. In addition, when we co-transfected the HCC cells with plasmids expressing YAP and those expressing uc.134, the decrease in the mRNA level of CYR61, c-Myc, diap1, and E2F1 was reversed by YAP, indicating that YAP is responsible for the uc.134-mediated gene regulation. Finally, a positive correlation between uc.134, LATS1, and pYAP^S127^ was confirmed in 90 paraffin-embedded samples by ISH and IHC. Taken together, our results suggest that uc.134 increased Hippo kinase activity and repressed the downstream target genes of YAP by inhibiting CUL4A-mediated ubiquitination and degradation of LATS1. To the best of our knowledge, this study is the first to investigate the critical role of lncRNAs in the regulation of LATS1 in HCC. Thus, this lncRNA may offer a promising approach for HCC therapy by inhibiting YAP and activating Hippo kinase signaling.

Previous studies have clarified that the major risk factor for HCC in China is hepatitis B virus (HBV) infection, although hepatitis C virus infection and exposure to toxic chemical substances also contribute to the incidence of HCC. The lncRNA MEG3 is regulated by miR-29a in a methylation-dependent, tissue-specific manner, and it contributes to the growth of HCC [20]. Histone deacetylase 3 (HDAC3) is involved in the suppression of HCC-related lncRNA-LET [19]. The aberrant expression of lncRNA uc.134 in HCC may be mediated by HBV infection at the transcriptional level, post-transcriptional level, or by epigenetic regulations such as DNA methylation or histone deacetylation [50]. In our study, we report that the ultraconserved lncRNA uc.134 suppressed the progression of HCC by inhibiting CUL4A-mediated ubiquitination of LATS1. Hippo kinase signaling is highly conserved among diverse species and regulates tissue overgrowth and development [51]. The classic tumor suppressor miRNA let-7 is also conserved among species and promotes cell cycle exit and cell differentiation both during normal development and in cancer [52, 53]. However, does uc.134 have a similar developmental expression pattern as the miRNA let-7? It will be interesting to unravel the underlying mechanisms that control the expression of uc.134 in both cancer and normal development in the future.

## Conclusions

Taken together, our data indicated that the expression of a novel lncRNA, uc.134, was repressed in HCC specimens and the expression of uc.134 was significantly correlated with the overall survival of patients. Notably, our results showed that the overexpression of uc.134 suppressed HCC cell proliferation, invasion, and metastasis in vitro and in vivo. In addition, we demonstrated that uc.134 plays a crucial role in activating Hippo kinase signaling by inhibiting CUL4A ubiquitination of LATS1 and increasing YAP^S127^ phosphorylation. Furthermore, we confirmed that uc.134 repressed the downstream target genes of YAP. Finally, ISH and IHC showed positive relationships among uc.134, LAST1, and pYAP^S127^ in 90 paraffin-embedded samples. Consequently, these results identified that lncRNA uc.134 activates Hippo kinase signaling by blocking CUL4A, suggesting that it may serve as a tumor suppressor and prognostic biomarker in HCC.

## Additional files

Additional file 1: Supplementary raw data 1. LncRNA expression profiling data analysis by lncRNA microarray. (XLS 2275 kb)

Additional file 2 Supplementary raw data 2. Differentially mRNA expression profiling by mRNA microarray. (XLSX 5963 kb)

Additional file 3: Supplementary raw data 3. The full GO data of mRNA microarray. (XLS 336 kb)

Additional file 4: Oligonucleotide sequences and primers for this study. (PDF 100 kb)

Additional file 5: Figure S1. Expression levels of six lncRNAs by qRT-PCR in HCC cells. (A) nc-HOXA11-86 (B) ENST00000426547 (C) ENST00000442971 (D) NR_027250 (E) ENST00000400856 (F) ENST00000394079. *P < 0.05; **P < 0.01; and ***P < 0.001. **Figure S2.** Full-length of human lncRNA uc.134 gene cloning. (A) The sequencing of PCR products from the 5′-RACE and 3′-RACE procedures showed the boundary between the universal anchor primer and lncRNA uc.134 sequences. (B) Nucleotide sequence of the full-length human lncRNA uc.134 gene. **Figure S3.** Representative images of FISH detecting endogenous lncRNA uc.134 molecules (green) in HCC cells. Nucleus (blue) was stained with DAPI. **Figure S4.** Transfection efficiency in HCC cells was assessed by qRT-PCR. All experiments were performed in triplicate, and results are presented as mean ± SD. *P < 0.05, **P < 0.01, and ***P < 0.001. **Figure S5.** Knockdown of uc.134 promotes the progression and invasion of HCC in vitro and in vivo. (A) Growth curves of HCC cells generated by CCK8 proliferation analysis. The mean ± SD is shown for five independent experiments. ***P < 0.001. (B) The colony formation assays (left panels); analysis of the number of colonies (right panels). All experiments were performed in triplicate, and results are presented as mean ± SD. ***P < 0.001. (C) Transwell assay for the indicated cells. All experiments were performed in triplicate, and results are presented as mean ± SD. ***P < 0.001. (D) Representative images of the scratch wound-healing assay. All experiments were performed in triplicate, and results are presented as mean ± SD. ***P < 0.001. (E) Tumors formed by cells transfected with EGFP-LV-shRNA targeting uc.134 were markedly larger and grew faster than tumors formed by control cells. (*n* = 5) *P < 0.05. (F) Lung metastasis model generated by injecting cancer cells into the tail veins of mice. HE staining shows the number and volume of lung metastases in each group (*n* = 6) **P < 0.01. **Figure S6.** The histologic examination of tumors. Upper panels: ISH staining, middle panels: KI67 staining, lower panels: HE staining. **Figure S7.** LATS1 and pYAP^S127^ expression levels in HCC specimens. (A, B) IHC analysis in 90 primary clinical specimens showed that the expression levels of LATS1 and pYAP^S127^ were significantly depressed in tumors compared with adjacent tissues. (C, D) Lower expression levels of LATS1 and pYAP^S127^ were related to the poor survival of patients. (PDF 5721 kb)

## Abbreviations

CHX: Cycloheximide
HCC: Hepatocellular carcinoma
ISH: In situ hybridization
lncRNAs: Long noncoding RNAs
MS: Mass spectrometry
RACE: Rapid amplification of complementary DNA ends
RIP: RNA-binding protein immunoprecipitation
WHDD: Winged helix-turn-helix DNA-binding domain
