# Correction: A novel lncRNA uc.134 represses hepatocellular carcinoma progression by inhibiting CUL4A-mediated ubiquitination of LATS1

**DOI:** 10.1186/s13045-023-01447-z

**Published:** 2023-05-08

**Authors:** Wen Ni, Yuqin Zhang, Zetao Zhan, Feng Ye, Yonghao Liang, Jing Huang, Keli Chen, Longhua Chen, Yi Ding

**Affiliations:** 1 Department of Radiation Oncology, Nanfang Hospital, Southern Medical University, Guangzhou, 510515 China; 2 Department of Pathology, Southern Medical University, Guangzhou, 510515 China; 3 Cancer Center, Affiliated Hospital of Guangdong Medical University, Zhanjiang, 524001 China

**Correction: Journal of Hematology & Oncology (2017) 10:91** 10.1186/s13045-017-0449-4

The original article [1] contains an error in Fig. 2C whereby there was mistaken overlap introduced in the preparation of the Bel7402 and Bel7402 control images. The corrected figure can be viewed in this correction article.

**Fig. 2:**
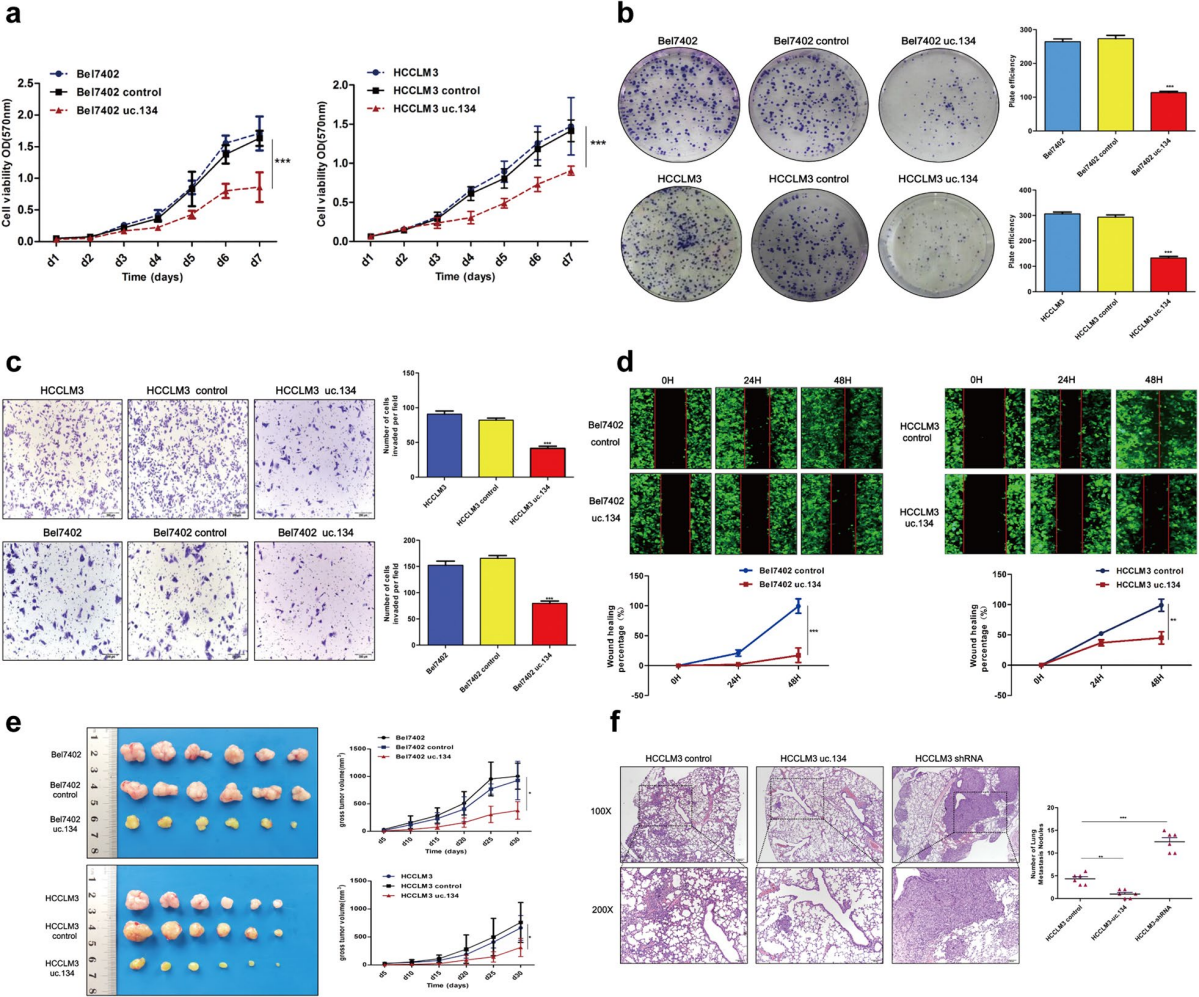
LncRNA uc.134 suppresses the proliferation, migration, and invasion of HCC in vitro and in vivo. **a** Growth curves of indicated HCC cells that overexpressed uc.134 compared with those of negative control cells and untransfected cells were determined with a CCK8 proliferation analysis. The mean ± SD is shown for five independent experiments. ***P < 0.001. **b** Representative images of colony formation assays (*left panels*); analysis of the number of colonies (right panels). All experiments were performed in triplicate, and results are presented as mean ± SD. ***P < 0.001. **c** Invasion ability of the indicated *cell lines* by Transwell assays. All experiments were performed in triplicate, and results are presented as mean ± SD. ***P < 0.001. **d** Representative images of the scratch wound-healing assay. The wound-healing percentage was analyzed with the ImageJ software. All experiments were performed in triplicate, and results are presented as mean ± SD. **P < 0.01 and ***P < 0.001. **e** Representative images of tumors formed in nude mice (*n* = 6). *P < 0.05. **f** Lung metastasis model generated by injecting tumor cells into the tail veins of mice. HE staining showing the number and volume of lung metastases in each group (*n* = 6); **P < 0.01 and ***P < 0.001
