# Supplementary material for: A novel lncRNA uc.134 represses hepatocellular carcinoma progression by inhibiting CUL4A-mediated ubiquitination of LATS1
Source: J Hematol Oncol. 2017 Apr 19;10:91. doi: 10.1186/s13045-017-0449-4 (PMC5395742; doi:10.1186/s13045-017-0449-4)
Supplement: Supplementary file 4 — Oligonucleotide sequences and primers for this study. (PDF 100 kb) [file 13045_2017_449_MOESM4_ESM.pdf]

Oligonucleotide sequences and primers for this study

qPCR primers gene expression and RIP: lncRNA uc.134 (forward, CCTTTAAGATCAGAGCAC; reverse, ACTTTCATCATAACCACC), nc-HOXA11-86(forward, CTGAGCCTTACGCTTCTTTC; reverse, CGCCTGGACCCACTTTATT), ENST00000426547(forward, ATCATCTCCCAAGAACAA; reverse, TTACAGCAATCCCAAGTC), ENST00000442971(forward, CATCACCTAGCGTCTTCG; reverse, :TGTTCTGACTGCTCCACC), ENST00000400856(forward, AGCCATCCTAAGAAACCT; reverse, ACTAGCAGCACTGACCCT), ENST00000394079(forward, GTTCCTGCTTTCACAGAATT; reverse, TTGCCATCCAACCACTCA), NR_027250 (forward, GAAGGGAGTGTCAGAAGC; reverse, AGAATGGCACAGTGTAAGA), YAP(forward, TAGCCCTGCGTAGCCAGTTA; reverse, TCATGCTTAGTCCACTGTCTGT), CUL4A(forward, ACCTCGCACAGATGTACCAG; reverse, AGGTTGACGAACCGCTCATTC), MST1(forward, CCTTGGTGCTACACAACAGAC; reverse, CAGACCTTGGTCGAGGAACTT), LATS1(forward, AATTTGGGACGCATCATAAAGCC; reverse, TCGTCGAGGATCTTGGTAACTC), CYR61(forward, CTCGCCTTAGTCGTCACCC; reverse, CGCCGAAGTTGCATTCCAG), c-Myc(forward, GGCTCCTGGCAAAAGGTCA; reverse, CTGCGTAGTTGTGCTGATGT), E2F1(forward,

ACGCTATGAGACCTCACTGAA; reverse, TCCTGGGTCAACCCCTCAAG), β-actin(forward, GGGAAATCGTGCGTGACATTAAG; reverse, TGTGTTGGCGTACAGGTCTTTG), GAPDH(forward, AGCTGAACGGGAAGCTCACT; reverse, TGCTTAGCCAAATTCGTTG).

The gene-specific primers used for the PCR of the RACE analyses were as follows: 5' RACE-outer: CGGACTTGGACGGCTTGCACAC;

5' RACE-inner: AGAAGCGGCTGCCCAGGTCGTT;

3' RACE-outer: GCCAGCCAGCAACAAAAGAG;

3' RACE-inner: TAATGAACACTAAAATGGGGAAGG.

siRNA: si-uc.134-1(sense, CCAUCCUCUAAUAAACCAATT; antisense, UUGGUUUAUUAGAGGAUGGTT), si-uc.134-2(sense, GAGACAGUAAACCUGGGUATT; antisense, UACCCAGGUUUACUGUCUCTT), si-LATS1-1(sense, GGGCAUGAAAUCCCUACAUTT; antisense, AUGUAGGGAUUUCAUGCCCTT), si-LATS1-2(sense, GCAGCGUCUACAUCGUAAATT; antisense, UUUACGAUGUAGACGCUGCTT), si-CUL4A-1(sense, GCAAAGCAUGUGGAUUCAATT; antisense, UUGAAUCCACAUGCUUUGCTT), si-CUL4A-2(sense, GCAAACUACUUUGGGACAUTT; antisense,

AUGUCCCAAAGUAGUUUGCTT).

LNA™ ISH probes: Scramble (/5DigN/GTG TAA CAC GTC TAT ACG CCC A/3Dig_N/), lncRNAuc.134 (/5DigN/TGTGCTCTGATCTTAAAGGCA/3Dig_N/).
