# Supplementary material for: A novel lncRNA uc.134 represses hepatocellular carcinoma progression by inhibiting CUL4A-mediated ubiquitination of LATS1
Source: J Hematol Oncol. 2017 Apr 19;10:91. doi: 10.1186/s13045-017-0449-4 (PMC5395742; doi:10.1186/s13045-017-0449-4)

Figure S1. Expression levels of 6 lncRNAs by qRT-PCR in HCC cells.

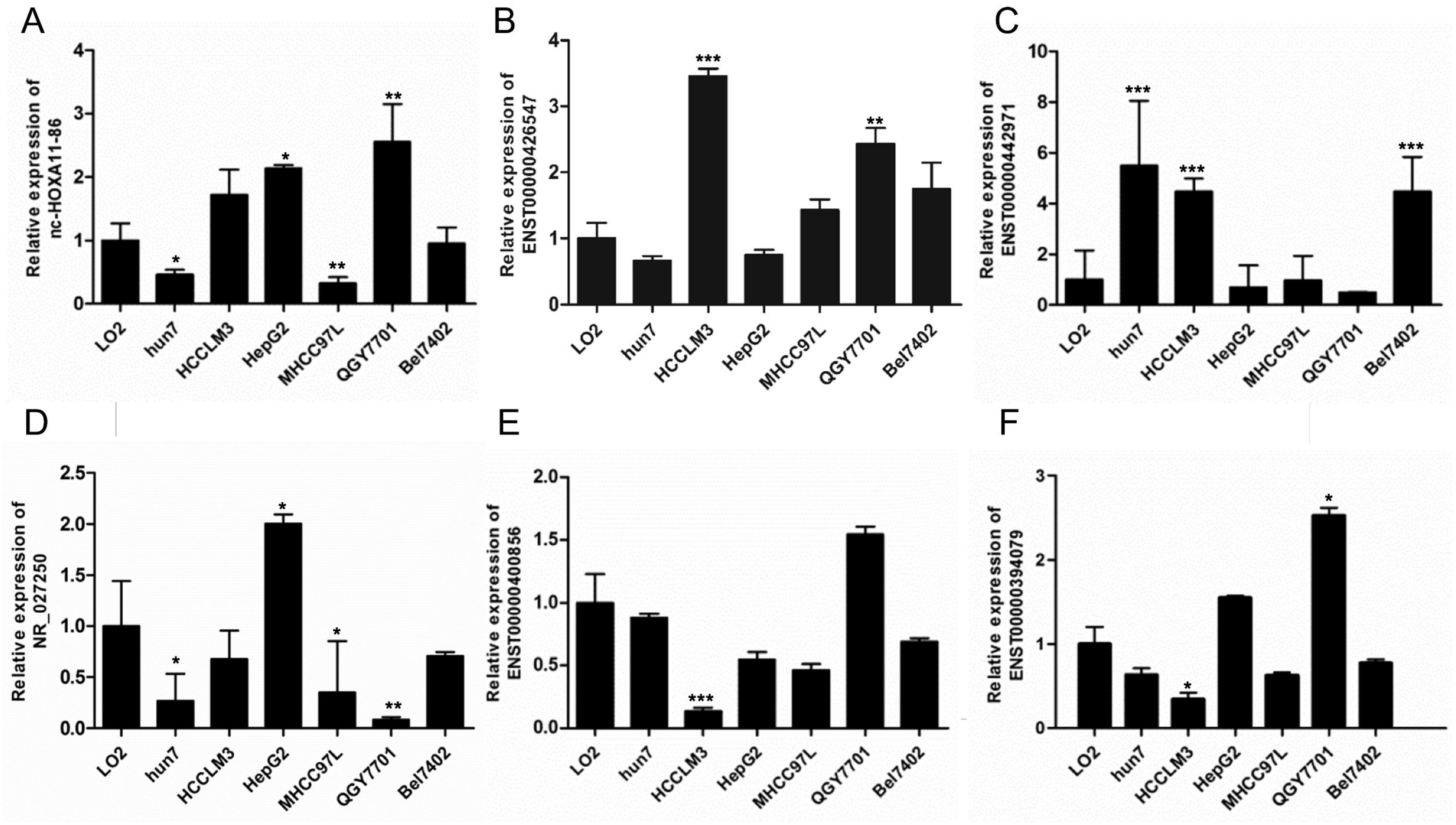

Figure S2. Full-length of human lncRNA uc.134 gene cloning.

A

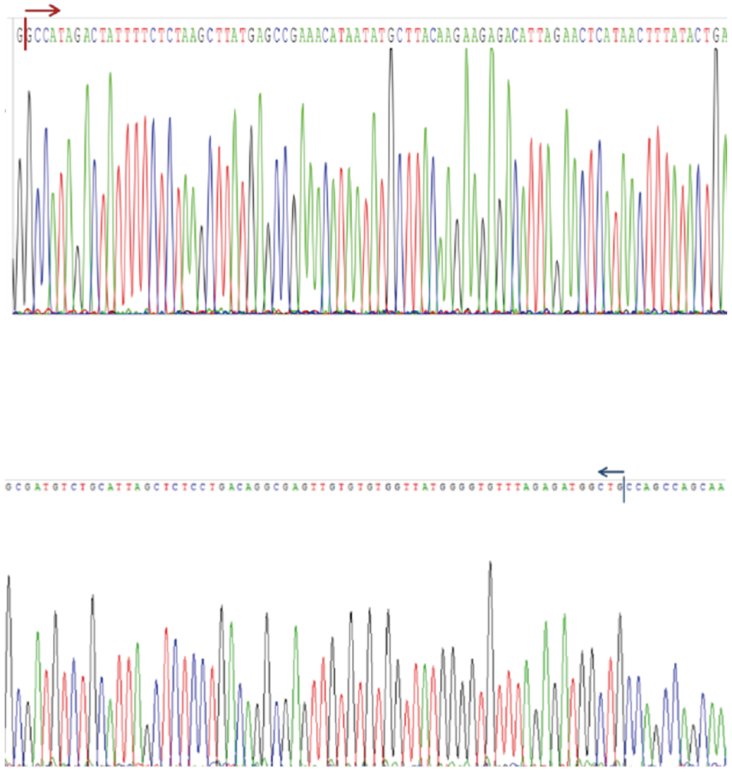

B

5’GCCATAGACTATTTTCTCTAAGCTTATGAGCCGAAACATAATATGCTTACAAGAAGAGAC
ATTAGAACTCATAACTTTATACTGACTAATGTTCAAATACAATTATGTTAGGTATCCATT
TGCTTCTGACATCACAAAAGAACAGATCACAAAAGACTTGGACTTAAGCTGTCTGCTTTT
CCTTGCATAATAAGTCCCCTGGTTCAGGGAATCCAGTCATTATAGAATTTAGCCTAACTG
CTTCTAATCCATGTAGGACTTTTTATTAAAGATGAAAACTAGTAAAGAATTAACCATGAG
AGGAAATAAAAGATTAATTCAAGTCAGTCATCACCGTATCTATATCTTAAGAAGTTAATA
GTTCAAAATCTTTTTTTTTTTTTTTTTTTTTTTACTGGTAGAAGACTTCTGAATATACTC
GCCGCTTCTGAATATACTCCCAGCCCCATGGTGTTAGGGGTAGGTTGCAACTATGTAACT
CCAAGCTGACTGGGCTGGCACCAACATCATCAGCACTGCTAACATCTTCCAGCAAGTTTT
AGTTCATTAATCTTAATGCCATACATGTACCCTTTCTCTAGCTAGTGGGTGGCCAGACAT
AAAAACTTGCAAACAGAGGCAAAGACATTAATGGGATGATGCAAGAAGAGAGTTGGAGAT
TTTTTTTTAAATCTCCTTTGCACATAGGAGTGACCCTAATCAATGCTTTTTGGTGAATAT
AAGCAGAATACATCTTGTTGCCTTCTCTAGAATAGAGAACTAAGTAGATATGTTGCCTCT
GGGTTCAATATTTATTTCTGGAAACTCTTTTCATTTTACTACCATCCTCTAATAAACCAA
TACATCCACTTTGTAAAATATAAGACTCTGGAGTTATTATATGTGCTTATAAAAACACAC
CTTTAGATAAGTGGTTTTTCCAATCTTCTGATACTCCTCATTTCCTTCTCATTGATTTTC
AAGTTTCAACACAATTTTTTAAAAATAAGGATTTAAACATGAGACAGTAAACCTGGGTAA
AGAACTAAGGGGTTTTGAAATTCAAAGTTATTATTTCTGTTTTTAGTAGCTGTTCCACCC
TTGCAAATATGTATAATAATTAGCCACAATTAATATTCCTCTTTTCCCTAATTCATTATA
AAAAGTATAACTTCTATACAATAATGCATTACATTTTTATCATGAGAAATCATAACAATA
TCTTTATATTTGAACCTGAACTTCAATATGAATTTGTGAGATTTTTTATATTAATGTGTG
TATTTTGTAATGTATGTGGTATGTGTATATATATGTTTATTTTAGACACATTTGACTACA
TTTATATATTCATAGAGATTTACATAGAGGCATTAAAAAAAACAGGCATAGTCAGTCATGG
ATGTTCATTATATATAAAGGTTATTGGGGATGCAAAGATATAAAAGATAGAAGAACCTAG
TAATTGCCTTTAAGATCAGAGCACAAAGCATTAGTTTGGATTTTACAAGGCCAGCCAGCA
ACAAAAGAGCAGTAATTTATGAGCTCAGCGTAAAAGAATAGCATAATTGCATTAAAAATT
AATGCTAATGAACACTAAAATGGGGAAGGTGGTTATGATGAAAGTTTGTATTCATCTTTC
CTGGTTTGAATGATGGCTAGTGGCTTGATTAATGGCTTAATTTCTATCAATTAGCGGGGA
AAAATCTCCTGCGAGACAAGGGTAAATTGAGCATTGAATTACATATCTTGCTTTTGTGTG
CTAAGAAAAAAGAGCTGCGTATCTCTGATATTAGCCACAGTGACAAATGTGGTGTGCCCT
GCGATGTCTGCATTAGCTCTCCTGACAGGCGAGTTGTGTGTGGTTATGGGGTGTTTAGAG
ATGGCTG**3’**

Figure S3. FISH detects endogenous lncRNA uc.134 molecules in HCC cells.

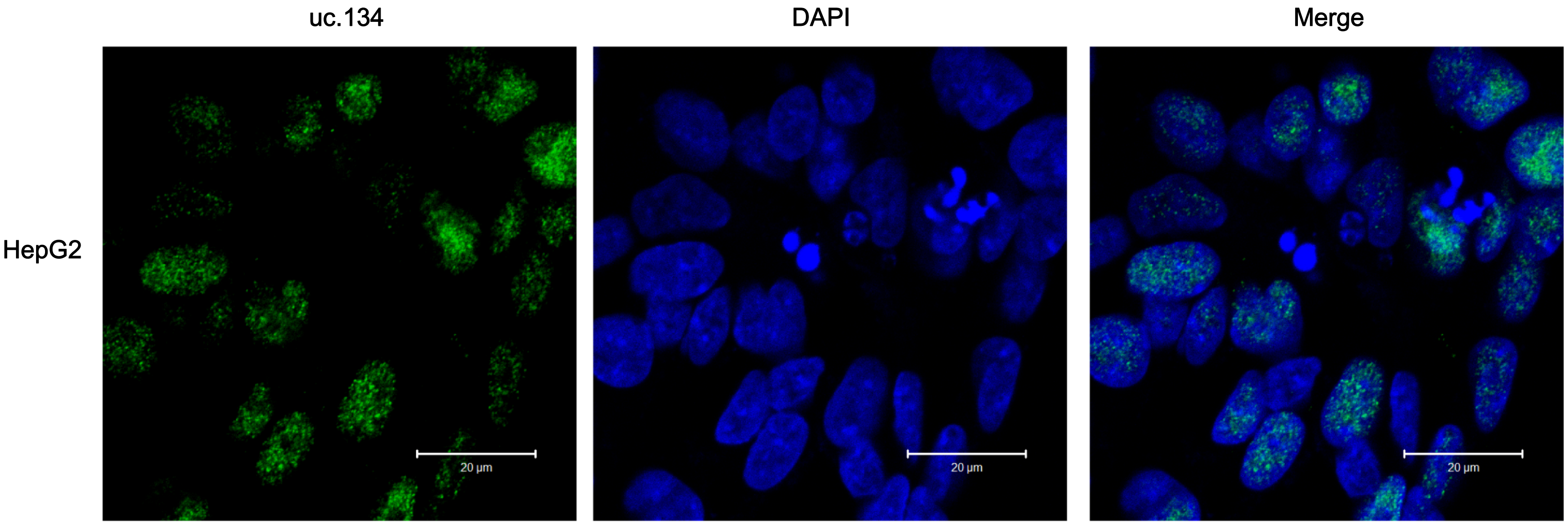

Figure S4. Transfection efficiency in HCC cells was assessed by qRT-PCR.

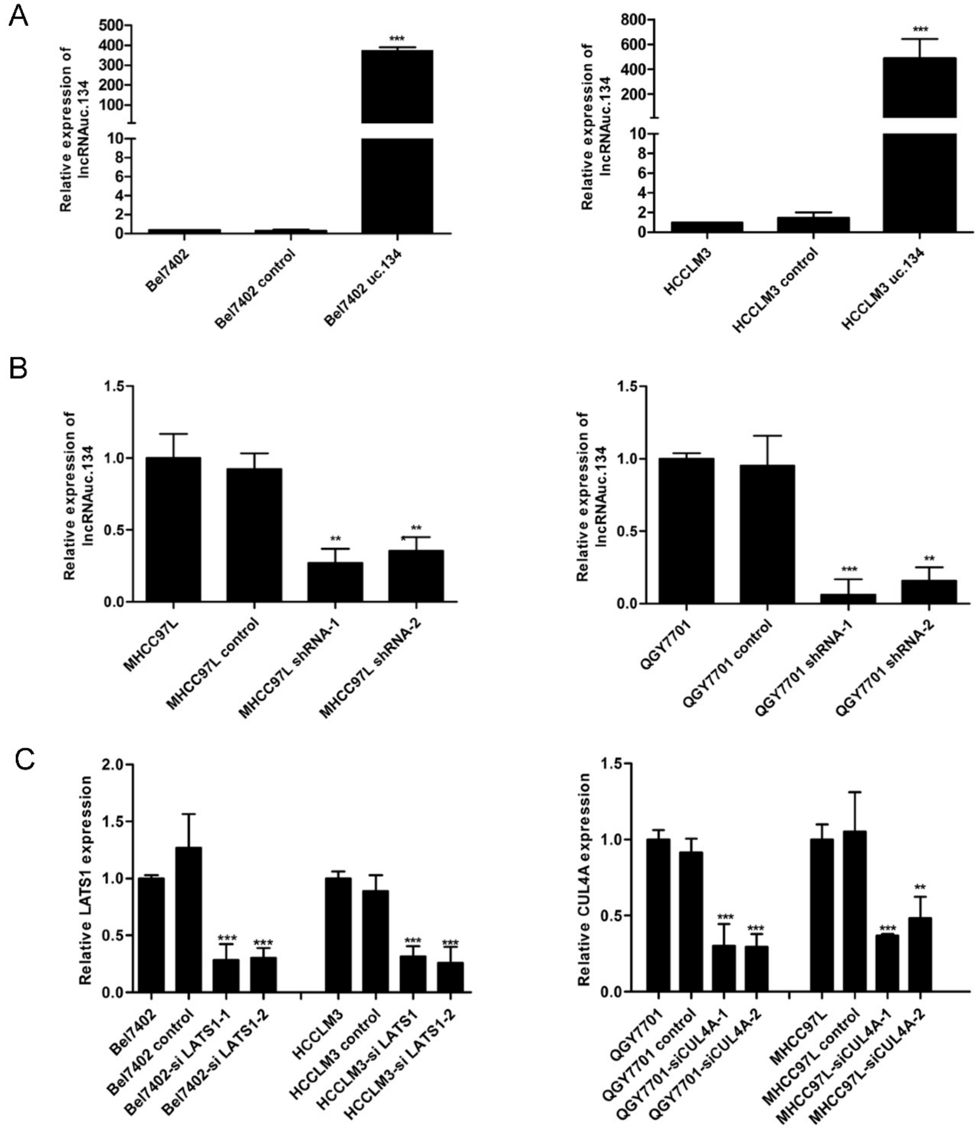

Figure S5. Knockdown of uc.134 promotes the progression and invasion of HCC in vitro and in vivo.

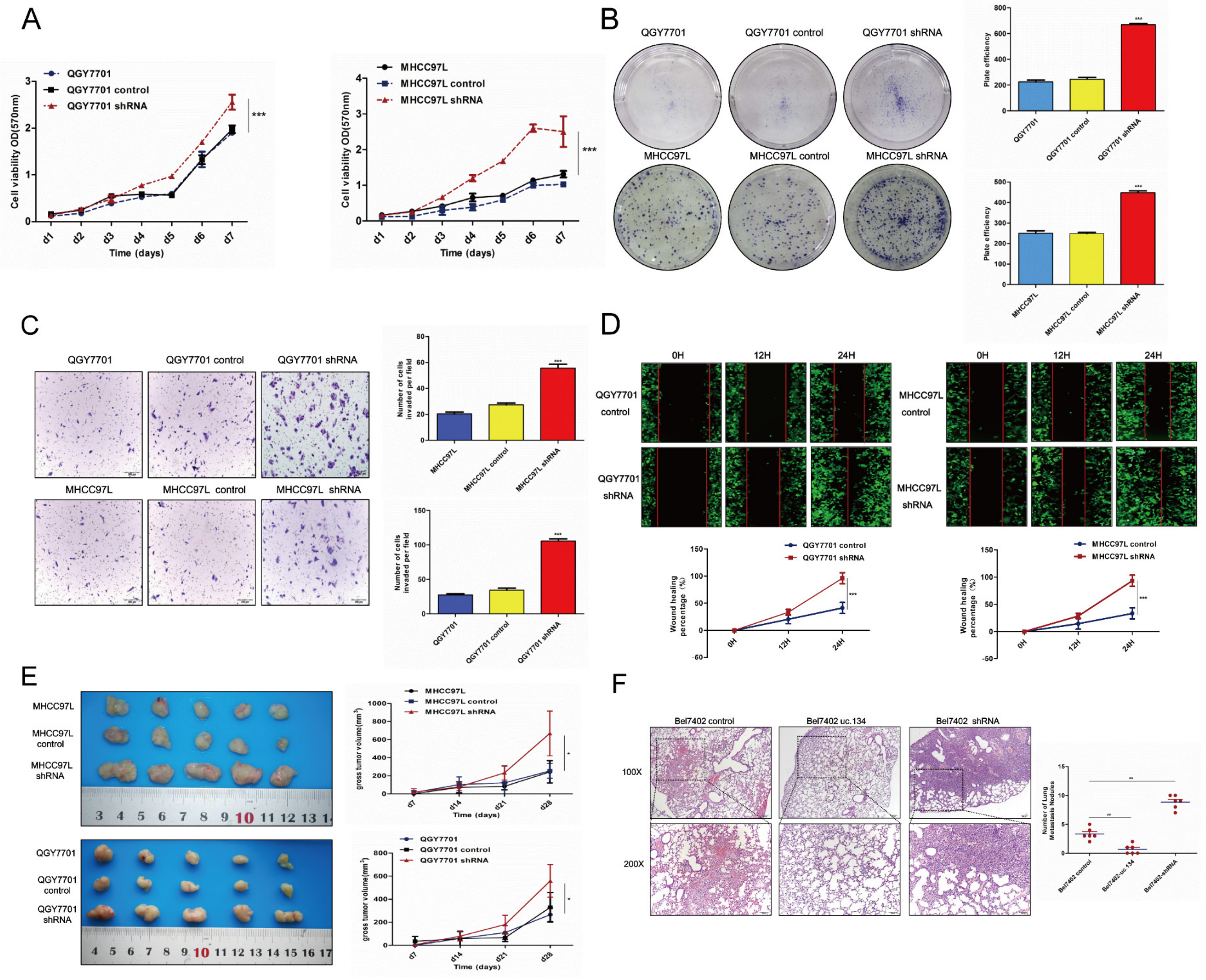

Figure S6. The histologic examination of tumors.

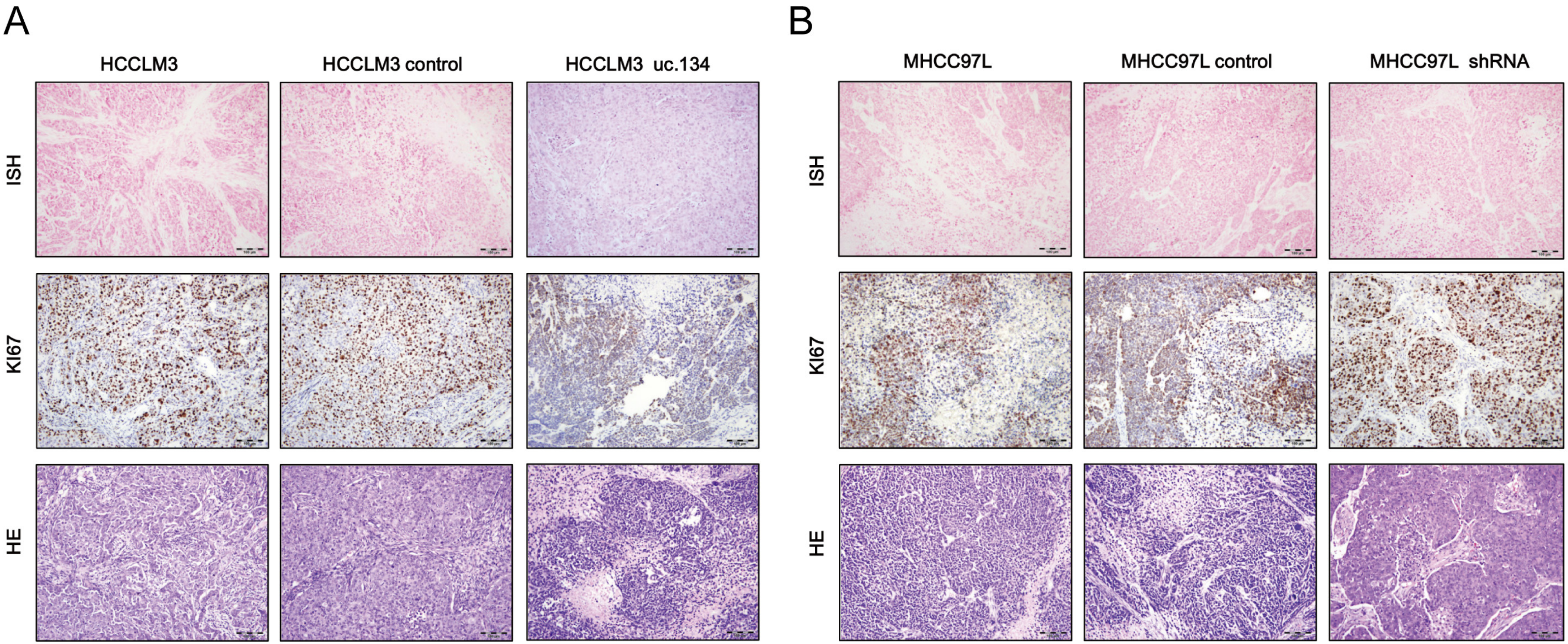

Figure S7. LATS1 and pYAP^{S127} expression levels in HCC specimens.

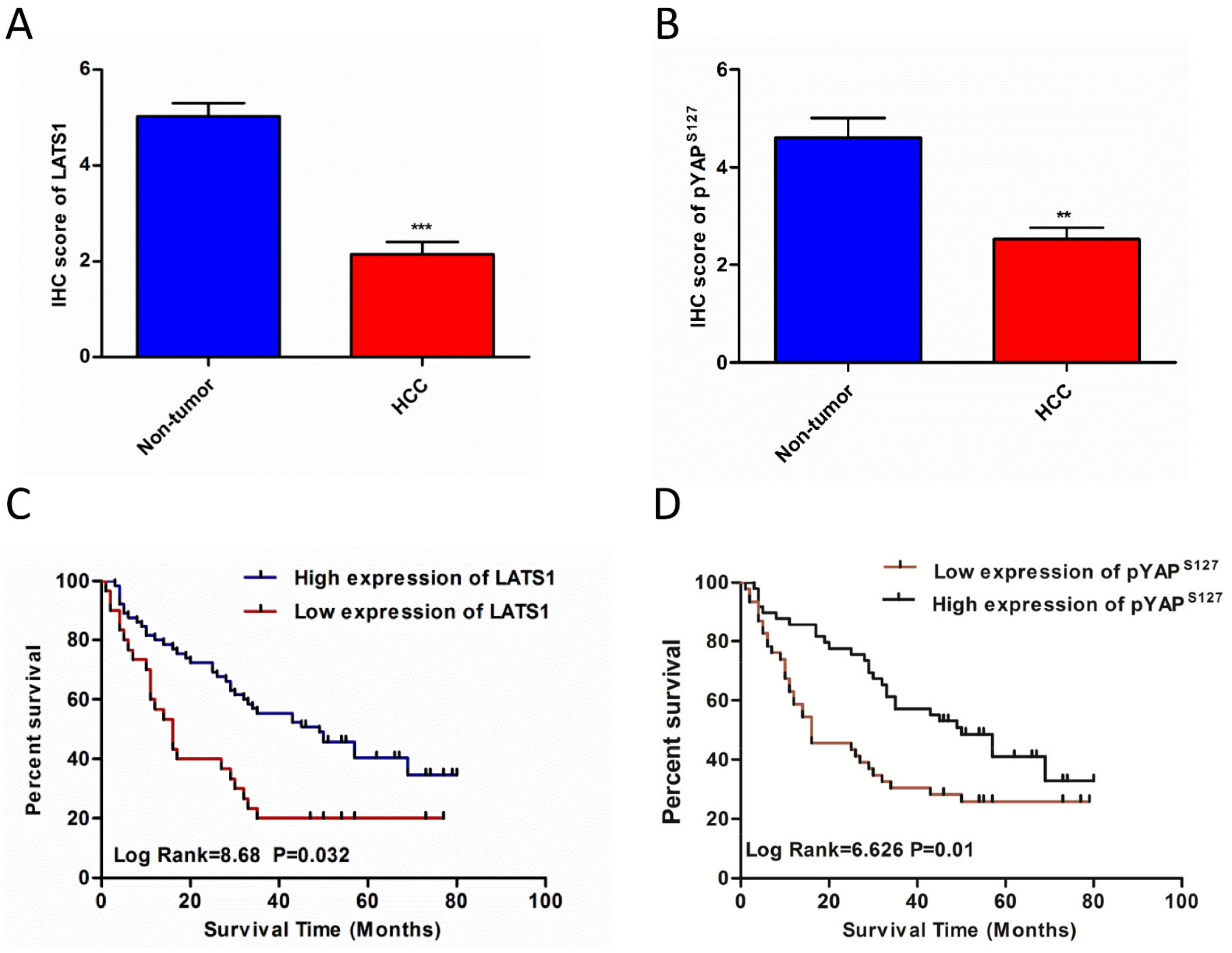

Supplement: Supplementary file 5 — Expression levels of six lncRNAs by qRT-PCR in HCC cells. (A) nc-HOXA11-86 (B) ENST00000426547 (C) ENST00000442971 (D) NR_027250 (E) ENST00000400856 (F) ENST00000394079. *P < 0.05; **P < 0.01; and ***P < 0.001. Figure S2. Full-length of human lncRNA uc.134 gene cloning. (A) The sequencing of PCR products from the 5′-RACE and 3′-RACE procedures showed the boundary between the universal anchor primer and lncRNA uc.134 sequences. (B) Nucleotide sequence of the full-length human lncRNA uc.134 gene. Figure S3. Representative images of FISH detecting endogenous lncRNA uc.134 molecules (green) in HCC cells. Nucleus (blue) was stained with DAPI. Figure S4. Transfection efficiency in HCC cells was assessed by qRT-PCR. All experiments were performed in triplicate, and results are presented as mean ± SD. *P < 0.05, **P < 0.01, and ***P < 0.001. Figure S5. Knockdown of uc.134 promotes the progression and invasion of HCC in vitro and in vivo. (A) Growth curves of HCC cells generated by CCK8 proliferation analysis. The mean ± SD is shown for five independent experiments. ***P < 0.001. (B) The colony formation assays (left panels); analysis of the number of colonies (right panels). All experiments were performed in triplicate, and results are presented as mean ± SD. ***P < 0.001. (C) Transwell assay for the indicated cells. All experiments were performed in triplicate, and results are presented as mean ± SD. ***P < 0.001. (D) Representative images of the scratch wound-healing assay. All experiments were performed in triplicate, and results are presented as mean ± SD. ***P < 0.001. (E) Tumors formed by cells transfected with EGFP-LV-shRNA targeting uc.134 were markedly larger and grew faster than tumors formed by control cells. (n = 5) *P < 0.05. (F) Lung metastasis model generated by injecting cancer cells into the tail veins of mice. HE staining shows the number and volume of lung metastases in each group (n = 6) **P < 0.01. Figure S6. The histologic examination of tumors. Upper [file 13045_2017_449_MOESM5_ESM.pdf]
